# Identification and characterization of mammaglobin-A epitope in heterogenous breast cancers for enhancing tumor-targeting therapy

**DOI:** 10.1038/s41392-020-0183-1

**Published:** 2020-05-28

**Authors:** Zhiqiang Liu, Xiqin Yang, Cuimi Duan, Jiangxue Li, Rongsheng Tong, Yuting Fan, Jiannan Feng, Ruiyuan Cao, Wu Zhong, Xiaoyan Feng, Heqiu Zhang, Lulu Cai

**Affiliations:** 1 Beijing Institute of Basic Medical Sciences, 27 Taiping Road, Haidian District, Beijing, 100850 China; 2 Personalized Drug Therapy Key Laboratory of Sichuan Province, Department of Pharmacy, Sichuan Provincial People’s Hospital, University of Electronic Science and Technology of China, Chengdu, 611731 China; 3 Beijing Institute of Pharmacology and Toxicology, 27 Taiping Road, Haidian District, Beijing, 100850 China

**Keywords:** Nanobiotechnology, Breast cancer, Target identification

## Abstract

Although targeted therapy has been extensively investigated for breast cancers, a molecular target with broad application is currently unavailable due to the high heterogeneity of these cancers. Mammaglobin-A (Mam-A), which is overexpressed in most breast carcinomas, has been proposed as a promising target. However, the lack of specific targeting moieties due to uncertain binding epitopes hampers further translational study. Here, seven potential epitopes of Mam-A were disclosed, and a unique epitope was then identified in most types of breast cancers, despite the genotypic heterogeneity. With phage display technology, the epitope was determined to be N-terminal amino acids 42–51 of Mam-A (N_42–51_). Then, the N_42–51_ epitope-specific monoclonal antibody, mAb785, was conjugated to poly lactic-co-glycolic acid (PLGA) nanoparticles loaded with therapeutic agents, thereby enhancing the drug uptake and therapeutic efficacy in different genotypes of breast cancers. The computer simulation of the N_42–51_ epitope and the mAb785 structures, as well as their interactions, further revealed the specific targeting mechanism of the mAb785-conjugated nanoparticles to breast cancers.

## Introduction

Breast cancer is the leading cause of cancer-related death in women.^1–3^ Currently, the most classic molecular targets for breast cancers include human epidermal growth factor receptor 2 (HER-2/neu), oestrogen receptor (ER), and progesterone receptor (PR).^4–7^ However, due to the heterogeneous molecular alterations in the breast cancer cells, patients with highly aggressive triple-negative breast cancer (TNBC) cannot benefit from HER-2/ER/PR-targeted therapies.^8,9^ The development of new targeting strategies, particularly for TNBCs, is urgently needed. Mammaglobin A (Mam-A) was reported to be highly specific to the mammary gland and upregulated in most mammary carcinomas of different genotypes, including TNBCs^10-12^ and it has been proposed as a promising molecular target for breast cancer therapy.^12,13^ However, despite a few scattered studies, a robust targeting system based on Mam-A is still unavailable due to the lack of understanding of its immune-epitopes and the lack of a proper ligand with high affinity.

A growing number of nano-drugs has been approved for clinical use, with the potential to revolutionize anti-cancer drug delivery.^14^ Nevertheless, the tumor-targeting effect of most approved nanocarriers is based on the enhanced permeation and retention (EPR) effect, which often leads to modest improvement in efficacy and sometimes unexpected side effects compared to current frontline therapies.^15^ On the other hand, many nanoparticle-based therapeutics, such as stimuli-responsive nanoparticles (NPs), are confined to the preclinical stages because of their complicated architecture for industrial processes or their undesirable tumor-targeting potency in vivo, which hampers their clinical translation.^16,17^ Thus, novel approaches to design nanomedicines with selective tumor binding and scale-up preparation processes have been in the spotlight and extensively investigated.^18–20^ The epitope is the part of the antigen that the antibody recognizes and binds to. Epitope-based approaches have been widely used to develop vaccines, antibody–drug conjugates, and CAR T cells for infection and cancer treatment.^21–24^ The identification of appropriate epitopes for targeting cells and the development of powerful components to bind to those epitopes would increase safety and enhance the therapeutic potency.^25^ Therefore, epitope-targeted nanomedicine would be of untapped clinical value for precise cancer therapy.

Herein, we established a novel targeting system for breast cancer nanomedicine, which was modified with a Mam-A-specific antibody, that could target the Mam-A immunoepitope. It acted like a “skeleton lock-and-key” system, overcoming the tumor heterogeneity. First, a mAb pool against Mam-A was established, by which the potential epitopes of Mam-A were systemically screened. Secondly, several membrane-associated epitopes and the corresponding mAbs were experimentally determined and one was revealed as the optimal epitope that was immunoactive in most breast cancers. Through phage display, the epitope was determined to be located at the N-terminal amino acids 42–51 (N_42–51_ aa) of Mam-A. Furthermore, the strof the epitope, as well as its interaction with the corresponding mAb, was confirmed by computer simulation. Mam-A presented a triangle-like fold, and N_42–51_ aa was located at the most prominent apex of Mam-A, which was easily gripped by the mAb. Finally, the optimal mAb–epitope targeting strategy was applied to nanomedicine by decorating the mAb on the surface of PLGA NPs that had been approved by the FDA. The mAb-modified PLGA NPs could target four genotypes of breast cancers and be uptaken by the tumor cells in vitro, significantly enhancing drug delivery as well as the anti-cancer efficacy against multiple breast cancers in vivo.

Using the entire sequence of the human Mam-A protein as an immunogen, we built tens of anti-Mam-A mAbs and screened 19 mAbs with superior affinity (Table S1). The epitope curve of Mam-A was mapped, and the possible distribution of epitopes within the protein was predicted using BioSun software.^26^ Based on this, Mam-A was cloned into four polypeptide fragments that contained different epitopes (Fig. S1a; polypeptides A, B, C, and D). Through the mAb–antigen reaction, six different epitopes in Mam-A and the corresponding mAbs were identified (Fig. S1b, Table S1). Meanwhile, several commercial mAbs against Mam-A were also evaluated, and only one (Thermo Scientific, Clone 304-1 A5, mAb304 for short) was found to react with polypeptide D, which was different from all the mAbs we had constructed. mAb304 was also the only one used for Mam-A targeting in previous publications.^10,13^ In total, seven different characteristics of the mAb–antigen reaction were observed, and, thus, all of these epitopes were identified within Mam-A.

**Fig. 1:**
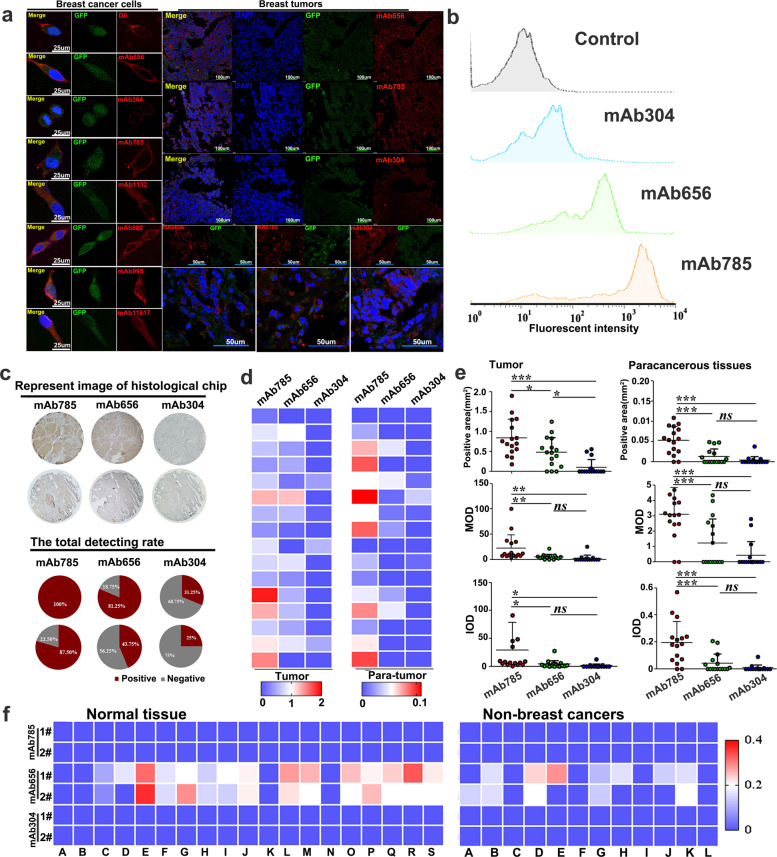
Screening and evaluation of membrane-associated anti-Mam-A mAbs. **a** ZR75.1 cells and tumor slides expressing green fluorescent protein (GFP) in the cytoplasm were immunostained by representative mAbs, targeting seven epitopes of Mam-A, and the DiI dye was used as a positive control of membrane staining. **b** Flow cytometry analyzing the fluorescent intensities of ZR75.1 cells immunostained by mAb785, mAb656 and mAb304. **c** Representative image of tissue chips stained by different mAbs and the total detecting rate. **d** Heat maps of Mam-A expression on breast cancer tissue chips stained by different mAbs. **e** Quantitative analysis of immunostained breast tumor samples on chips by different mAbs (*P < 0.05, **P < 0.01, ***P < 0.001, One way ANOVA /Tukey’s multiple comparisons test was used for multiple group comparison). **f** Evaluation of mAb specificity by tissue chips, including non-breast cancer tissue and normal tissue chips. MOD, mean optical density; IOD, integrated optical density

For tumor targeting, it is essential for the intended molecular target to be accessible on the surface of the cancer cells.^27^ For this, immunofluorescent staining of the breast cancer cell ZR75.1, a Mam-A high-expressing cell line,^28^ was chosen to determine the membrane-associated epitopes among the seven epitopes mentioned above. ZR75.1 cells expressing green fluorescent protein (GFP) in the cytoplasm were used, and control cells were stained with membrane dye DiI. As shown in Fig. 1a, mAb656 and mAb785 were observed with membrane-specific staining on breast cancer cells, besides the previously reported mAb304. Moreover, membrane-specific staining by mAb785, mAb656, and mAb304 were further verified at tumor tissue levels.

**Fig. 2:**
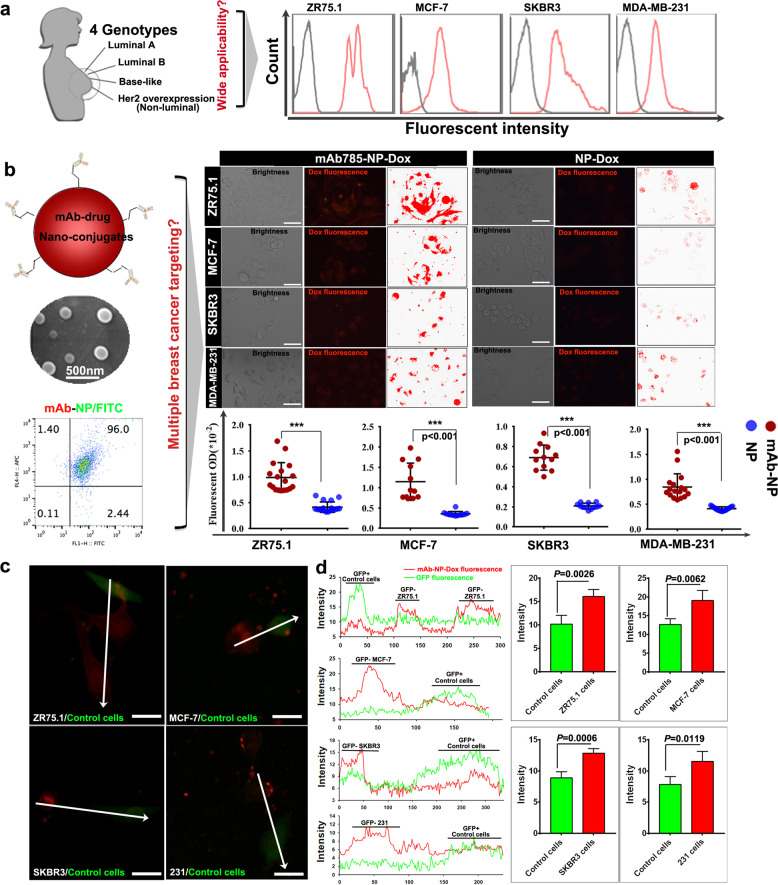
The efficacy of mAb785 as ligand mediating the targeting of NPs to multiple breast cancers. **a** The targeting of mAb785 to four genotypes of breast cancer cells. **b** Preparation and SEM image of mAb785-NPs, confirmation of the successful conjugation of mAbs onto the NPs by flow cytometry, and the determination of targeting capacity of mAb785-NPs to multiple breast cancer cells by fluorescent microscope (Bar = 100 μm, Two-tailed Student's *t* test was performed for statistical analysis). **c** Multiple breast cancer cells (ZR75.1, MCF-7, SKBR3, and MDA-MB-231) were cocultured with non-breast cancer cells (control cells, GFP positive). mAb-NP-Dox (Red fluorescence) was incubated with cocultured cells and the relative red fluorescent intensities were measured along the white arrowheads (Bar = 50 μm). **d** intensity distribution of red and green fluorescence which indicated the distribution of red fluorescent signals were opposite to that of green fluorescent signals, that was, GFP+ cells were detected with relative low red fluorescence while GFP− cells were detected with relative high red fluorescence (two-tailed Student's *t* test was performed for statistical analysis)

The targeting efficacy of the three membrane-associated mAb–epitope systems mentioned above was systemically analyzed at molecular, cellular, and histological levels, respectively. At the molecular level, ELISA results showed that the mAb785 had the highest affinity compared to mAb656 and mAb304 (Supplementary Fig. S1c, d). At the cellular level, the highest fluorescent intensity was also detected in mAb785-immunostained ZR75.1 cells by immunostaining and flow cytometry (Fig. 1b and Supplementary Fig. S1e). The above results were further confirmed with surface plasmon resonance (Supplementary Fig. S2, Supplementary Table S2). For histological analysis, chips including 16 cases of breast cancer and the corresponding paracancerous tissues were customized and immunostained with mAb785, mAb656, and mAb304. All 16 breast cancer cases were positively detected by mAb785 (13 cases by mAb656 and 5 cases by mAb304), and consistent results were achieved from 16 paracancerous samples (Supplementary Fig. S3; Fig. 1c). The results from the tissue chips were expressed as thermograph (Fig. 1d). Statistical analysis showed that positive areas on the chips stained by mAb785 were larger than those stained by mAb304 and mAb656 (Fig. 1e), and the mean optical density (MOD), as well as the integrated optical density (IOD), on chips stained by mAb785 were obviously higher than those stained by mAb304 and mAb656. Mam-A expression in breast cancers has been investigated by different groups, with an upregulation rate of 40% to more than 90%.^11,29,30^ The above results from different mAb suggest that the variation may be ascribed to epitope-specific antibodies. Some epitopes of Mam-A might be shielded or inactivated under certain forms against antibody binding, and thus, they could not be detected by epitope-specific antibodies.^31^ To evaluate the specificities of the above three mAbs, chips including 19 types of normal tissue (two samples for each type;Supplementary Fig. S4) and chips including 12 types of tumor and paracancerous tissues (Supplementary Fig. S5) were customized. Nonspecific staining on several normal or non-breast cancers was observed for mAb656, while none was observed for either mAb785 or mAb304 (Fig. 2f; Supplementary Table S3; Supplementary Table S4). Collectively, the above results indicate that mAb785 and its epitope are sensitive and specific system that may be applicable in breast cancer-targeted therapy.

To demonstrate the potential of the mAb785–epitope system for breast cancer targeting, representative breast cancer cells from four different genotypes were selected, including ZR75.1 (luminal A subtype), MCF-7 (luminal B subtype), SKBR3 (Erb-B2 overexpression subtype), and MDA-MB-231 (basal-like subtype). Flow cytometry demonstrated that all four breast cancer cell lines could be positively targeted by mAb785 (Fig. 2a). Then, the efficacy of the targeting system was applied to engineer nanomedicine (Fig. 2b) by conjugating mAb785 to the surface of the PLGA NPs (Supplementary Fig. S6a, b) since PLGA has been approved as a biodegradable polymer by the FDA.^32,33^ The conjugation was verified by immunofluorescent staining and flow cytometry (Supplementary Fig. S6c, Fig. 2b).

**Fig. 3:**
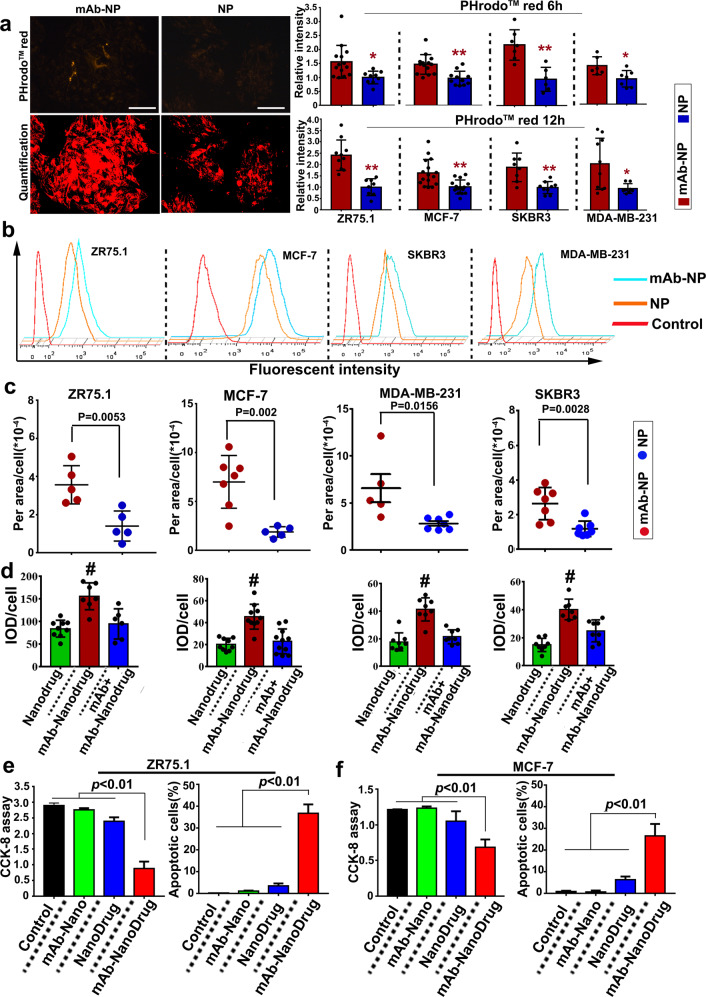
mAb785–epitope interactions promoted the uptake of NPs by breast cancer cells. **a** Phagocytosis indicator (pHrodoTMred)-conjugated Zymosan bioparticles were mixed with mAb785-conjugated or plain NPs. The mixed particles were incubated with four genotypes of breast cancer cells, and the uptake activities were quantified by measuring phagocytosis indicator fluorescence after 6 h and 12 h (*P < 0.01, **P < 0.01, Bar = 200 μm, Two-tailed Student's *t* test was performed for statistical analysis). **b** The phagocytosis indicator fluorescence was further quantified by flow cytometry. **c** Coumarin-loaded (green) NPs were mixed with pHrodoTM red, and the uptake of coumarin was quantified by co-locating green and red fluorescence (two-tailed Student’s *t* test was performed for statistical analysis). **d** The uptake of the nanodrug carrying FAM-labeled siRNA (targeting survivin) and Dox were quantified by the fluorescent signals (# *P* < 0.05 compared to the other groups, One way ANOVA/Tukey’s multiple comparisons test was used for statistical analysis). **e**, **f** mAb785-conjugated nanodrug-mediated inhibition of breast cancer cells ZR75.1 and MCF-7

To determine the targeting capacity, FITC-incorporated NPs (with and without mAb785 conjugation) were incubated with ZR75.1 and MCF-7 cells, respectively. As shown in Fig. S7, significant binding of mAb-NPs with breast cancer cells was detected. We further explored the feasibility of this targeting system for the delivery of doxorubicin (Dox) to four genotypes of breast cancer cells. Significantly higher Dox fluorescence was detected in mAb785-NP-Dox-treated cells than in NP-Dox-treated ones, indicating the active targeting of mAb785NPs to breast cancer cells (Fig. 2b). We then investigated the specificity of mAb785NPs to breast cancers. GFP-labeled non-breast cancer cells were cocultured with different breast cancer cells (Supplementary Fig. S8). After incubation with mAb785-NP-Dox, the red fluorescence distribution in breast cancer cells (GFP−) vs control cells (GFP+) was measured (Fig. 2c and Supplementary Fig. S8). It was shown that the red fluorescence was mostly observed in the breast cancer cells (Fig. 2c, d), but little was observed in the non-breast cancer cells, indicating the specificity of mAb785NPs to breast cancers. In addition to targeting, uptake of NPs by cancer cells is also important for targeted nanomedicine.^34,35^ To determine the cellular uptake of mAb785-NPs, a phagocytosis indicator (pHrodo^TM^ red conjugated Zymosan bioparticles) was mixed with NPs with or without mAb785 modification, respectively. Zymosan bioparticles were similar to nanoparticles in size and therefore, when they were mixed together for incubation with cells, they would be phagocytized together. pHrodo^TM^ Red was a pH-sensitive fluorescent dye. Outside the cells where PH was neutral, nearly no fluorescence was observed; after internalization into cytoplasm where the PH was faintly acid, mild red fluorescence would be observed; When phagocytic vesicle was combined with lysosomes, where the acidity was further increased, relative strong red fluorescence would be observed. Thus, the fluorescent signal changes could be used as indicator of nanoparticle internalization (outside cells→ inside cells→ lysosome, Supplementary Fig. S9). As shown in Supplementary Fig. S10 and Fig. 3a, the mixed particles were incubated with four breast cancer cell lines. After incubation for 6 h and 12 h, mAb785-NP-treated cells were observed with significantly higher fluorescence than NP-treated cells, indicating the enhanced uptake of NPs through mAb785 binding, which was also verified by flow cytometry (Fig. 3b). It should be noted that after 6 h incubation, mild red fluorescence was detected in most cells, indicating that nanoparticles were phagocytized into cytoplasm and mainly in free state; after 12 h incubation, relative strong red fluorescence was detected in most cells, indicating that lots of phagocytized nanoparticles were combined by lysosome within cells. To directly determine the nanodrug uptake, coumarin with green fluorescence was encapsulated in NPs for labeling. Co-localization of green and red fluorescence was identified and quantified as internalized nanodrugs. As shown in Supplementary Fig. S11 and Fig. 3c, more internalized coumarin were detected in mAb785-NPs than the plain NPs group, providing further evidence that mAb785 modification significantly potentiated the uptake of nanodrugs by different genotypes of breast cancer cells.

To determine the efficacy of the targeting system in breast cancer treatment, we prepared a multifunctional “Nanodrug” that encapsulated Dox inside itself and absorbed siRNAs (targeting surviving gene) outside itself (Supplementary Fig. S12) because Dox and siRNA could inhibit cancers synergistically (Supplementary Fig. S13).^36–38^ In addition, mAb785-NPs were coated with polyethyleneimine to improve the siRNA loading capacity (Supplementary Fig. S14). To determine the targeting capacity of siRNA-carrying NPs, Cy5-labeled siRNA was loaded onto mAb785-NPs or plain NPs. As shown in Supplementary Fig. S15, Cy5 imaging demonstrated the targeted binding of siRNA-loaded mAb785-NPs with breast cancer cells. Then, we determined the efficacy of gene silencing and found that mAb785-NPs loaded with siRNA were more efficient than plain NPs and commercial liposome (Supplementary Fig. S16). Furthermore, mAb785-NPs were incorporated with NH_3_HCO_3_ to acquire pH responsiveness so as to promote the intracellular release of Dox (Supplementary Fig. S17).^33^ To evaluate the targeting and uptake of the Nanodrug by multiple breast cancers, FAM-labeled siRNAs (green fluorescence) and Dox (red fluorescence) were co-loaded. After incubation with cancer cells, increased fluorescence (yellow as co-localization of red and green) was detected in mAb785–Nanodrug-treated cells (Fig. 3d, Supplementary Figs. S18, S19), indicating the enhanced delivery and uptake of Nanodrugs by mAb785 modification.

**Fig. 4:**
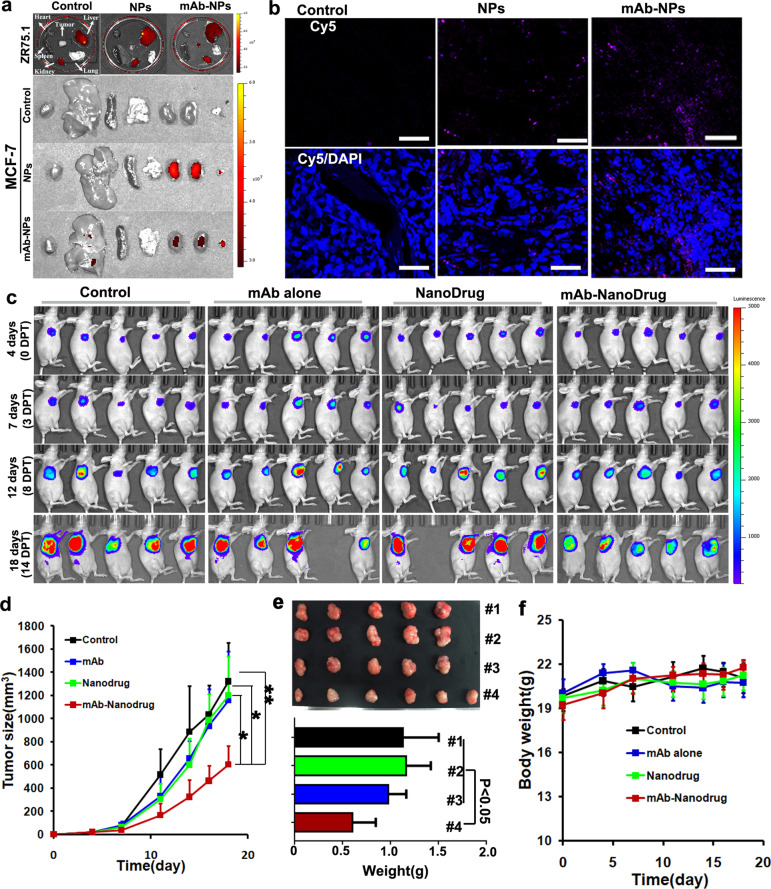
mAb785-conjugated nanodrugs targeted and inhibited breast cancers in vivo. **a** Near-infrared imaging of the distribution of mAb785–Nanodrugs in organs from mice with breast tumor. **b** Fluorescent microscopy of mAb785–Nanodrugs in tumor sections (ZR75.1 tumor). **c** Bioluminescent imaging of tumor growth in mice treated with mAb785–Nanodrugs. **d** Tumor growth curve of different groups (*P < 0.05; **P < 0.01; One way ANOVA /Tukey’s multiple comparisons test was used for statistical analysis). **e** Tumor weight at the end of the in vivo experiment (one way ANOVA/Tukey’s multiple comparisons test was used for statistical analysis). **f** The body weight of animals

To determine the anti-cancer efficacy, two breast cancer cell lines, ZR75.1 and MCF7, were evaluated in vitro. The CCK8 assay and TUNEL staining consistently showed that mAb785–Nanodrugs significantly inhibited cell proliferation compared with the Nanodrug alone (Fig. 3e, f; Supplementary Fig. S20). Meanwhile, to determine the specificity of the mAb785-Nanodrug to breast cancers, an irrelevant cancer cell A549 which does not express Mam-A was used to evaluate anti-cancer efficacy in vitro. CCK8 assay demonstrated slight inhibition of A549 cells by nanodrugs, which may be due to the non-specific adsorption of nanodrugs to the cells (Supplementary Fig. S21). No significant difference was detected between nanodrug-treated cells and mAb785–Nanodrug-treated ones, indicating the lack of specific response to mAb785–Nanodrug for the irrelevant cancer cell. For in vivo evaluation, nude mice bearing breast tumors were prepared using ZR75.1 and MCF-7 cells. After injection through the caudal vein, the targeting of mAb785–Nanodrug to tumors in vivo was confirmed by NIR imaging of Cy5-labeled siRNAs in the tissues (Fig. 4a) and fluorescent microscopy of tumor sections (Fig. 4b). RT-PCR analysis of the tumors also demonstrated that the survivin gene was significantly inhibited in mAb785–Nanodrug-treated animals (Supplementary Fig. S22). Bioluminescent imaging showed that MCF-7 tumor (labeled with luciferase) growth was significantly inhibited in the mAb785–Nanodrug group compared to the control, mAb785 alone, and the Nanodrug groups (Fig. 4c and Supplementary Fig. S23a). Measurement of tumor growth was consistent (Fig. 4d) with bioluminescent imaging. At the end of the experiment, weighting of tumors also demonstrated the significant tumor inhibition by mAb785–Nanodrug (Fig. 4e). The anti-cancer efficacy of mAb785–Nanodrug was also confirmed in models bearing the ZR75.1 tumors (Supplementary Fig. S23b). Meanwhile, the body weight was measured during the experiment and it demonstrated no significant difference among different groups (Fig. 4f), which may suggest that no significant toxicity of mAb785–Nanodrug existed. To further explore the potential toxicity of mAb785–Nanodrug, several main organs including heart, liver, spleen, lung, and kidney were analyzed by H&E staining, and no significant damage was observed in any organs (Supplementary Fig. S24), providing additional evidence for the little toxicity of the Nanodrug.

To further confirm the efficacy of the targeting system in multiple breast cancers, TNBC models were prepared using MDA-MB-231 cell line. 24 h after delivery, the distribution of Cy5-labeled Nanodrugs in TNBC tumors was detected by fluorescent microscope. As shown in Supplementary Fig. S25, obviously more mAb-conjugated Nanodurgs were detected in tumor sections than control Nanodrugs, indicating the in vivo targeting of mAb-conjugated nanodrugs to TNBC tumors, which was consistent to in vitro results. The results suggested that using mAb785 antibody could also enhance the drug delivery into TNBC tumors. However, in the following experiment, no significant inhibition of TNBC tumors was detected in vivo (Data not shown). We supposed that it may be due to the following reasons: (1) the expression level of Mam-A was lower in MDA-MB-231 cells than those in ZR75.1 and MCF-7 cells. Thus, the bond nanodrug dose may be lower in TNBC tumors in vivo than those in ZR75.1 and MCF-7 tumors, which was confirmed too by in vitro experiments (Figs. 2b, 3c); (2) MDA-MB-231 cells may be more tolerant to drugs used in the study (doxorubicin and siSurvivin) than ZR75.1 and MCF-7 cells. We supposed that significant efficacy on TNBC using the present targeting system may be achieved by employing highly toxic or TNBC-specific antitumor drugs, which deserved further investigation in future.

**Fig. 5:**
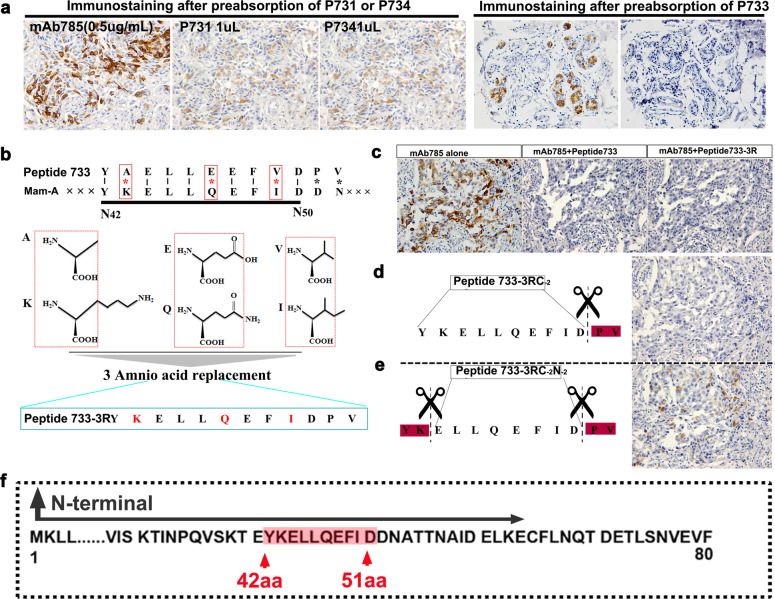
Location of mAb785 targeted epitope determination and computer-assisted structure reconstruction. **a** Three enriched phages were used for the pre-blocking test. Only phage 733 (P733) could completely block mAb785 immunostaining in breast cancer sections (Bar = 100 μm). **b** The three variant amino acids in peptides carried by phage 733 were highly similar to those in the Mam-A protein. **c** Replacing the three variant amino acids in the peptides from phage733 did not influence their reactivities with mAb785 (Bar = 100 μm). **d** Removing the last two amino acids in the peptides did not influence their reactivities with mAb785. **e** Removing the first two amino acids in the peptides significantly influenced their reactivities with mAb785. **f** The epitope for mAb785 in Mam-A was determined as the N-terminal 42–51 amino acids

**Fig. 6:**
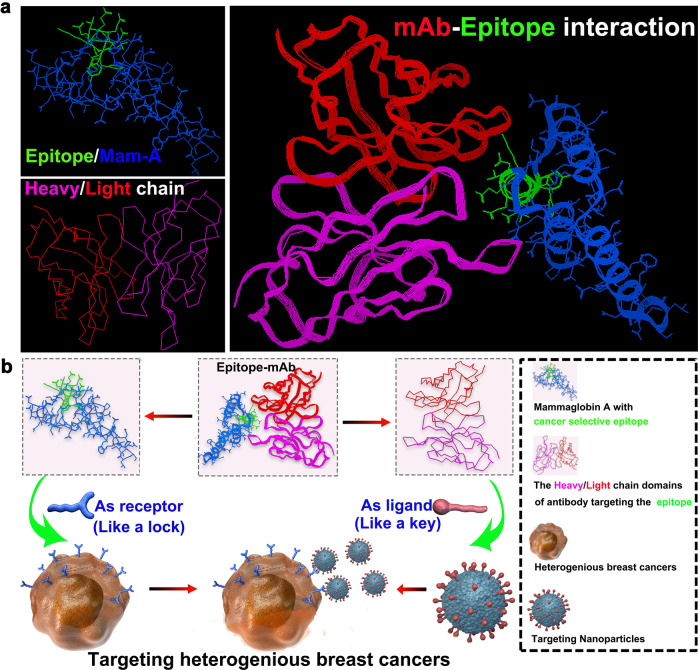
The underlying mechanisms of the general targeting system overcoming the heterogenicity of breast cancers. **a** Computer-assisted resolution of the structures of the epitope, Mam-A, and the viable region of mAb785, as well as their interactions. **b** Schematic illustration of enhanced drug delivery by the mAb785–Nanodrugs for heterogenous breast cancer-targeted therapy

The detailed location and sequence of the epitope were determined by phage display technology (Supplementary Fig. S26). Three phage colonies (P731, P734, and P733) were screened with a higher affinity to mAb785 (Supplementary Table S5). The pre-blocking test in the ELISA assay showed that the mAb785–antigen reactivity could be partially blocked by P731 and P734, while it could be completely blocked by P733 (Supplementary Fig. S27). The results were further confirmed by immunostaining of breast cancer sections (Fig. 5a), suggesting that the peptides carried by P733 may be the target epitope of mAb785. The peptides were sequenced as YAELLKEFVDPV (Supplementary Table S5). Sequence alignment demonstrated that the first 10 amino acids of the peptide (total 12 aa) were mostly the same as the N_42–51_ aa of Mam-A. More importantly, three different pairs of amino acids (AAs) were similar in property and they were mutually substitutable (Fig. 5b), suggesting that the N_42–51_ aa of Mam-A could be the target epitope of mAb785. For verification, we synthesized Peptide 733 (carried by P733) and Peptide 733-3R (three substitutable AAs were replaced with the same AAs in the primary Mam-A). Pre-blocking with the Peptide 733 and Peptide 733-3R both totally blocked mAb785 immunostaining of the breast cancer sections (Fig. 5c). To determine whether the last two AAs (PV) were necessary for the reaction with mAb785, a truncated peptide without PV was synthesized (Peptide 733-3RE_-2_). It was found that Peptide 733-3RE_-2_ and Peptide 733-3R blocked mAb785 in the same way, suggesting that the last two amino acids (PV) were unnecessary for the epitope (Fig. 5d). To determine the epitope sequence more precisely, we further truncated the first two AAs from Peptide 733-3RE_-2_ and obtained peptides 733-3RE_-2_F_-2_. The pre-blocking test showed that mAb785 could not be completely blocked by Peptide 733-3RE_-2_F_-2_ (Fig. 5e), indicating that the first two AAs in Peptide 733-3RE_-2_ were essential for the epitope. The above results were further verified by the ELISA assay (Supplementary Fig. S28). Collectively, the epitope of mAb785 was determined as YKELLQEFID and located at N_42–51_ within the Mam-A protein (Fig. 5f). Based on the location and sequence, the structure of the epitope within Mam-A was computationally reconstructed. The variable region of mAb785 was also computationally reconstructed after antibody sequencing (Supplementary data: Antibody sequences of mAb785). Furthermore, the interaction between mAb785 and the epitope was computationally simulated (Fig. 6a). Mam-A presented as a triangle-like fold, and the N_42–51_ aa was located at the most salient and stable vertex of Mam-A, which could make the epitope easily exposed and recognizable by its antibodies. The variable region of mAb785 folded into a puller-like structure and perfectly fit with the N_42–51_ epitope. The computer-assisted simulation of the N_42–51_ epitope and mAb785 structures, as well as their interactions, could explain the mechanism underlying the specific targeting of the mAb785-NPs to breast cancers, as illustrated in Fig. 6b.

The study successfully screened a specific targeting system for heterogeneous breast cancers based on the Mam-A epitope. When applied to nanomedicine, this system allowed for the active targeting of antibody-conjugated NPs to multiple breast cancers. Importantly, the intracellular uptake of NPs by multiple breast cancer cells was significantly enhanced due to the epitope–antibody interaction. Consequently, the anti-cancer efficacies of the drug-loaded NPs were significantly augmented against different genotypes of breast cancers. Furthermore, the precise targeting site was determined as the N_42–51_ AAs of Mam-A. The structures and interactions of the epitope with its optimal antibody were further revealed by computer simulation. Understanding the location and sequences of the epitope could extend the epitope-based approach for targeted cancer therapy, e.g., developing new CART cells. Collectively, the immunoepitope–mAb785 acted like a “skeleton lock-and-key” system, opening the doors between nanomedicine and multiple breast cancers, which could potentially be a tool for targeted drug delivery to heterogeneous breast cancers.

## Materials and methods

### Preparation of anti-human mammaglobin-A (Mam-A) monoclonal antibodies

The procedures for the immunization, preparation, and selection of hybridomas cells, as well as the collection and purification of monoclonal antibodies (mAbs) are described in our previous report.^11^

### Screening potential epitopes in Mam-A

The epitope curve of Mam-A was mapped using BioSun Version 3.0 software (The Center of Computational Biology, Beijing Institute of Basic Medical Sciences, China), as our previous reports.^11,26^ Based on this, Mam-A was cloned into four polypeptide fragments that could contain different epitopes (polypeptides A, B, C, and D; see Fig. S1). Indirect ELISA was performed to screen effective mAbs that could react with Mam-A or the polypeptides.

### Immunofluorescent staining and flow cytometry

Cells were fixed with cold methanol/acetone and incubated with mAbs. For laser scanning confocal microscopy, AF594-labeled anti-mouse IgG antibodies (Abcam, USA) were used as secondary antibodies. Nuclei were stained by DAPI. DiI staining was performed according to the manufacturer’s instructions (Invitrogen). For flow cytometry, AF647-labeled anti-mouse IgG antibodies were used as secondary antibodies. The stained cells were analyzed using a flow cytometer (FACSCalibur, BD, USA).

### Surface plasmon resonance

Surface plasmon resonance was performed using a Biacore 3000™ instrument (GE Healthcare, USA). CM5 sensor chips, a thiol ligand coupling kit, and HBS–EP + 10× running buffer (0.1 mol/L Hepes, 1.5 mol/L NaCl, 30 mmol/L ethylene diamine tetraacetic acid [EDTA], and 0.5% [v/v] p20) were all purchased from GE Healthcare.

### Cell targeting and uptake assay

To evaluate cell targeting, cells were incubated with NPs for 2 h and then washed with PBS. The binding of the NPs to the cancer cells was observed under a fluorescent microscope according to the carried fluorescent tag. For the cell uptake assay, breast cancer cells were incubated with a mixture of phagocytosis indicator pHrodo^TM^ Red conjugated Zymosan bioparticles (Thermo Fisher Scientific, P35364) and prepared NPs. The cellular uptake was evaluated according to the fluorescent signal of the phagocytosis indicator. The quantification of fluorescent signals was performed using flow cytometry or Image-Pro Plus software.

### Cell viability and apoptotic assay

The cell viability and apoptosis were evaluated using CCK-8 staining (CCK-8 staining kit, Dojindo, Japan) and TUNEL staining (TUNEL staining kit, Key GEN INC., China), respectively, using commercial kits according to the manufacturers’ instructions.

### Phage display screening

A phage display library containing 10^11^ random peptides (12 amino acids) was used (Biolabs, Lot:0411702). Monoclonal antibodies were coated onto an ELISA plate to capture phages; un-captured phages were removed by washing. Captured phages were eluted for expansion. The expanded phages were used for the second capture by antibodies. With the same protocol, a total of 3 phage expansion-capture cycles were repeated to enrich high-frequently captured phages. Then, the phages were sequenced to determine the peptide sequences.

### Pre-blocking test, antibody sequencing, and computer-assisted structure analysis

For the pre-blocking test, antibodies were pre-incubated with phages or peptides for blocking and then used for the ELISA reaction or immunostaining. Antibody variable domain sequencing was performed by GenScriptInc. Based on the epitope and antibody sequences, the immunoreactive structure of the epitope in Mam-A and the mAb variable region were computationally calculated and simulated using Insightll 2000 software.

### Preparation and characterization of the nanodrugs

Poly (lactic-co-glycolic) acid (PLGA, MW = 100,000; lactide/glycolide = 50:50) was purchased from Shandong Institute of Medical Instrument (Jinan, China). PLGA NPs were prepared using the emulsion–evaporation method.^32,33^ For details, see the supplementary material.

### Animal experiments

All experimental protocols related to animals were approved by the Animal Care Committee of the Beijing Institute of Basic Medical Sciences and the Ethics Committee of Sichuan Provincial People’s Hospital, China. To prepare nude mice bearing breast tumors in situ, 5 × 10^6^ breast cancer cells in 100 μL of PBS were injected into the mammary fat pad of the nude mice. Intraperitoneal injection of oestradiol benzoate (100 μg) was performed twice a week. One week after cell injection, the animals were randomly divided into different treatment groups. The control group was injected with 100 µL of PBS into the caudal vein. Nanodrugs containing 20 μg of Dox and 100 pmol of siRNA in 100 μL of PBS were injected through the caudal vein four days after cell seeding. The length (*L*) and width (*W*) of the tumors were measured with a calliper, and the tumor size was calculated as *L* × *W* × *W*/2, as in previous reports.^39^

### Non-invasive optical imaging

Bioluminescent and near-infrared imaging with a highly sensitive charge-coupled device (CCD) camera (IVIS50, Xenogen, USA) was performed. For bioluminescent imaging, D-Luciferin (150 mg/kg body weight) was intraperitoneally injected into nude mice, and then the animals were anesthetised by inhaling isoflurane (2–3%). The imaging was performed for 1–10 min until the maximum signals were obtained. Near-infrared imaging of Cy5 was performed to detect the distribution of NPs in animals and tissues.

### Statistical analysis

Student’s *t* test has been used for two groups’ comparison, and the One way ANOVA has been used for more than two groups’ comparison. All data are expressed as the mean ± SD. Statistical analysis was performed using SPSS 17.0, GraphPad Prism7, and OriginPro8. *P* < 0.05 was considered statistically significant. Multiple software packages were used for plotting, including Excel2007, OriginPro8, and GraphPad Prism7.

## Supplementary information

Identification and Characterization of Mammaglobin-A Epitope in Heterogenous Breast Cancers for Enhancing Tumor-Targeting Therapy
