# Supplementary material for: Identification and characterization of mammaglobin-A epitope in heterogenous breast cancers for enhancing tumor-targeting therapy
Source: Signal Transduct Target Ther. 2020 May 28;5:82. doi: 10.1038/s41392-020-0183-1 (PMC7256037; doi:10.1038/s41392-020-0183-1)
Supplement: Supplementary file 1 — Identification and Characterization of Mammaglobin-A Epitope in Heterogenous Breast Cancers for Enhancing Tumor-Targeting Therapy [file 41392_2020_183_MOESM1_ESM.doc]

**SUPPLEMENTAL INFORMATION**

Supplementary information contains:

Online methods

Supplementary tables: Table S1-S5

Supplementary figures and figure legends: Figure S1-S27 and legends

Antibody sequences of mAb785

**Identification and Characterization of Mammaglobin-A epitope in heterogenous breast cancers for enhancing tumor-targeting therapy**

*Zhiqiang Liu^*^,*†*^,1^, XiqinYang*†*^,1^, Cuimi Duan^1^, Jiangxue Li^1^,* *Rongsheng Tong^2^, Yuting Fan^2^,Jiannan Feng^3^, Ruiyuan Cao^3^, Wu Zhong^3^,Xiaoyan Feng^1^, Heqiu Zhang^*1^,Lulu Cai^*2^*

^1^Beijing Institute of Basic Medical Sciences, 27 Taiping Road, Haidian District, Beijing 100850, China.

^2^Personalized Drug Therapy Key Laboratory of Sichuan Province, Department of Pharmacy, Sichuan Provincial People's Hospital, University of Electronic Science and Technology of China, Chengdu 611731, China

^3^Beijing Institute of Pharmacology and Toxicology, 27 Taiping Road, HaidianDistrict, Beijing 100850, China.

†These authors contributed equally to this work

*Corresponding authors: Lulu Cai(cailulu@med.uestc.edu.cn); Heqiu Zhang (zhangheqiu2004@126.com); Zhiqiang Liu(zhiqiangliu_amms@163.com).

**Online methods**

**Preparation and characterization of Nanodrugs**

Poly (lactic-co-glycolic) acid (PLGA, Mw: 100,000;lactide: glycolide=50:50) was purchased from Shandong Institute of Medical Instrument (Jinan, China). PLGA nanoparticles (NPs) were prepared using emulsion-evaporation method^1, 2^. Briefly, PLGA was dissolved in dichloromethane to prepare a 6% oil phase solution. 1mL PLGA solution was poured into 10 ml of 2.5% PVA (Sigma) aqueous solution and then sonicated for 1min at 4% maximal power using an ultrasonic cell disruptor (SCIENTZ-IID) to obtain an oil-in-water emulsion. The emulsion was agitated for several hours at room temperature to allow for solventevaporation. The PLGA NPs were collected by centrifugation at 12000g for 10min. After washing with distilled water for 3 times, the PLGA NPs were lyophilized for preservation. To prepare doxorubicin (Dox) or FITC encapsulated NPs, Dox and FITC were co-dissolved in dichloromethane with PLGA. The loaded Dox was quantified by dissolving 10mg NPs in DMSO and measured by spectrofluorometer at 480 nm. PLGA NPs were suspended in distilled water and then dropped on the aluminum foil. After dried in the air, the samples were placed on a metal stub double-sided conductive adhesive tape and covered with a thin layer of gold using a sputter coater. The samples were then examined by a scanning electronic microscope (JSM-6301F scanning microscope). A light scattering particle sizer (MalvernZS90, Master sizer/E/Malvern, Inc.) was used to measure the size and zeta potential of NPs. For conjugation with mAbs, NPs were incubated with an appropriate amount of NHS (50 mM) and EDC (100mM) for 30 min. After washing with PBS, NPs was suspended in PBS and excess mAbs were added. The mAb was incubated with NPs at 4°C overnight. By washing with PBS for three times, the redundant mAbs were removed. To detect the successful conjugation of mAbs, NPs was incubated with AF647-labeled anti-mouse IgG after blocking by BSA. The NPs were observed under fluorescent microscopy or analyzed by flow cytometry. For non-covalent adsorption, mAbs were incubated with nanoparticles for 1 h，and the redundant mAbs were removed by washing with PBS for three times. DOX was encapsulated into particles during preparation. Antibodies were chemically conjugated to the surface of particles through EDC/NHS activation. The siRNAs were loaded outside after coating particles with 1% (w/v) polyethyleneimine^3^ (PEI, Mw: 25000Da, Sigma). To ensure the targeting capacity of nanoparticles *in vivo*, antibody-conjugated particles carrying Dox and siRNAs were further incubated with antibodies for ligand adsorption, BSA was used instead of antibodies for control nanoparticles. The siRNAs targeting survivin, 5ʹAAGCAUUCGUCCGGUUGCGCU3ʹ(Sense),5ʹAGCGCAACCGGACGAAUGCUU3ʹ (Antisense)were used.

**Cell culture**

Breast cancer cells ZR75.1, MCF-7, MDA-MB-231 and SKBR3， as well as non-breast cancer cell A549(lung cancer), were preserved in our lab. DMEM medium (Gibco) supplemented with 10% fetal bovine serum (FBS) was used for ZR75.1, MDA-MB-231, SKBR3 and A549 cells, while 1640 medium (Gibco) supplemented with 10% fetal bovine serum (FBS) was used for MCF-7 cells.

**Histology**

After sacrifice of animals, tumors and organs were explanted and fixed with 4% paraform. After dehydration by sucrose solution, tumors were embedded into O.C.T to prepare frozen sections. Organ samples, including hearts, livers, spleens, lungs and kidneys were dehydrated by gradient alcohol, treated by xylene and embedded into paraffin to prepare paraffin-embedded sections. For fluorescent microscope, nuclei was stained by DAPI solution. H&E staining was performed with routine procedures.

**RNA interference and RT-PCR**

RNA interference of the survivin gene was performed using PEI-coated NPs and mAb-NPs as carriers respectively. The nanoparticles carrying siRNA were added into OptiMEM (Gibco) for cell incubation. After 12-24h, the medium was replaced by fresh DMEM supplemented with 10% FBS. Liposome-mediated siRNA transfection using a commercial RNAiMAX kit (GenePharme, Shanghai, China) was performed for comparison according to the manufacturer’s instruction.

Total mRNAs from cells or tissues were extracted using the Trizol reagent (Life Technologies). The real-time PCR using SYBER Green Master Mix (Takara, Dalian, China) was performed with Applied Biosystems 7500 Fast Real-Time PCRSystem (Applied Biosystems, Carlsbad, USA). Primers for human survivin were as follows: forward: 5’-CAGATTTGAACGCGGGACCC-3’; reverse, 5’- CCAAGTCTGGCTC

GTTCTCAG-3’. The primers for GAPDH were as follows: forward, 5’-TGTGGTCATGAGTCCTTCCA-3’;reverse, 5’- ATGTTGGTCATGGGTGAGAA-3’.TheCt value was quantified with the 2^-ΔΔCt^ method and the relative level of survivin was normalized with respect to internal reference expression.

**Supplementary References**

1. Kishimoto, T. K. et al. Improving the efficacy and safety of biologic drugs with tolerogenic nanoparticles. *Nat nanotechnol.* 11, 890-899(2016).

2. Mitsunaga, M. et al. Cancer cell-selective in vivo near infrared photoimmunotherapy targeting specific membrane molecules. *Nat med.* 17, 1685-91(2011).

3. Jeon, S. Y.; Park, J. S.; Yang, H. N.; Woo, D. G.; Park, K. H. Co-delivery of SOX9 genes and anti-Cbfa-1 siRNA coated onto PLGA nanoparticles for chondrogenesis of human MSCs. *Biomaterials.* 33, 4413-23(2012).

**Supplementary tables**

**Table S1**.Preparation, characterization and screening of anti-Mam-A monoclonal antibodies

| **Clone number** | **IgG isoforms** | **Reactivity** | **Ascitic titer(*10^4^)** | **Concentration** |
| --- | --- | --- | --- | --- |
| D553 |  | Polypeptide A | 0.5 | 0.18mg/ml |
| 822 | IgG1 | Polypeptide A | 214.8 | 6.2mg/ml |
| MHH916 | IgG2a | Polypeptide A | 51.2 | 0.27mg/ml |
| MHG1152* | IgG2b | Polypeptide A | 12.8 | 0.36mg/ml |
| MJE1113 | IgG2a | Polypeptide A, B | 51.2 | 0.19mg/ml |
| MGD785* | IgG2a | Polypeptide A, B | 51.2 | 5mg/ml |
| CHH11617* | IgG2a | Polypeptide B | 204.8 | 1.5mg/ml |
| CGB161 | IgG1 | Polypeptide B | 12.8 | 0.95mg/ml |
| CFH931 | IgG1、IgG2a | Polypeptide B | 51.2 | 0.25mg/ml |
| 521 | IgG1 | Polypeptide B, C | 819.2 | 5.3mg/mL |
| CIA1072 | IgG1、IgG2a | Polypeptide B, C | 51.2 | 0.25mg/ml |
| CID611 | IgG2a | Polypeptide B, C | 51.2 | 0.58mg/ml |
| CHH995* | IgG2a | Polypeptide B, C | 51.2 | 0.68mg/ml |
| MJF656* | IgG1 | Polypeptide C | 51.2 | 1mg/ml |
| MHB584 |  | Polypeptide C | 0.8 | 0.22mg/ml |
| HF747 |  | Polypeptide C | - |  |
| IF923 |  | Polypeptide C | - |  |
| HF159 |  | Polypeptide C | - |  |
| CFE882* | IgG1 | Whole Mam-A | 51.2 | 1.11mg/ml |

*mAbMHG1152, MGD785, CHH11617, CHH995, mAb656,mAb882 were selected as representative mAbstargeting each epitope in the following experiment. They were termed mAb1152, 785, 11617, 995, 656 and 882 for short in the main text.

**Table S2.** Affinity constant of antibodies *

| **Affinity constant** | **ka (10^4^ 1/Ms)** | **kd (10^-4^ 1/s)** | **KD (10^-9^ M)** | **Chi^2^** |
| --- | --- | --- | --- | --- |
| **mAb785 to M** | 30.70±2.97 | 7.32±1.49 | 2.42±0.66***** | 34.10±9.15 |
| **mAb656 to M** | 0.51±0.09 | 3.01±0.12 | 55.20±6.65 | 13.80±6.35 |
| **mAb304 to M** | 0.99±0.02 | 3.25±1.22 | 33.00±13.00 | 10.70±1.27 |
| **mAb785 to A** | 10.60±0.59 | 0.15±4.51 | 10.70±3.66 | 5.93±0.77 |
| **mAb656 to C** | 0.43±0.00 | 0.91±0.61 | 21.30±14.30 | 2.80±0.42 |
| **mAb304 to D** | 1.79±0.10 | 5.37±0.30 | 30.10±3.19 | 21.60±1.42 |

* The values represent mean ± standard error.

**Table S3**. Reactivity with non-breast cancer tissues

| **Cancers** | **mAb785** | **mAb304** | **mAb656** |
| --- | --- | --- | --- |
| **Bladder** | **-** | **-** | **-** |
| **Esophagus** | **-** | **-** | **-** |
| **Stamoch** | **-** | **-** | **+** |
| **Colon** | **-** | **-** | **+** |
| **Kidney** | **-** | **-** | **-** |
| **Liver** | **-** | **-** | **-** |
| **Lung** | **-** | **-** | **+** |
| **Ovary** | **-** | **-** | **-** |
| **Prostate** | **-** | **-** | **+** |
| **Skin** | **-** | **-** | **+** |
| **Cervixuteri** | **-** | **-** | **-** |

“-”Indicated negative staining; “+” indicated positive staining

**Table S4**. Reactivity with normal tissues

| **Normal Tissues** | **mAb785** | **mAb304** | **mAb656** |
| --- | --- | --- | --- |
| **Breast** | **-** | **-** | **-** |
| **Cerebellum** | **-** | **-** | **-** |
| **Cerebral cortex** | **-** | **-** | **-** |
| **Oesopagus** | **-** | **-** | **+** |
| **Stomach** | **-** | **-** | **+** |
| **Small intestine** | **-** | **-** | **+** |
| **Colon** | **-** | **-** | **+** |
| **Heart** | **-** | **-** | **+** |
| **Kidney cortex** | **-** | **-** | **+** |
| **Liver** | **-** | **-** | **+** |
| **Lung** | **-** | **-** | **-** |
| **Ovary** | **-** | **-** | **+** |
| **Pancreas** | **-** | **-** | **+** |
| **Prostate** | **-** | **-** | **-** |
| **Skin** | **-** | **-** | **+** |
| **Testis** | **-** | **-** | **+** |
| **Thyroid** | **-** | **-** | **+** |
| **Tonsil** | **-** | **-** | **+** |
| **Endometrium** | **-** | **-** | **+** |

**Table S5** The enriched polypeptides and their frequency of occurrence

| **Phage clone** | **Sequence** | **Frequency** |
| --- | --- | --- |
| 731 | GRHSALLSEFWT | 5 |
| 733 | YAELLEEFVDPV | 5 |
| 734 | APLDMPYPRWFW | 2 |
| 735 | AHRTWNELLTEF | 1 |
| 736 | WLDLKAEFLQPP | 1 |
| 7311 | KVWPSPSMMFST | 1 |
| 7312 | KHSIHDNGPGFP | 1 |
| 7313 | HVAKISDTMLRD | 1 |
| 7315 | IPSTSVTATWSV | 1 |
| 7321 | FSPYALLEVHRA | 1 |
| 7322 | SVQYLRDSLVHY | 1 |

**Supplementary figures and figure legends**


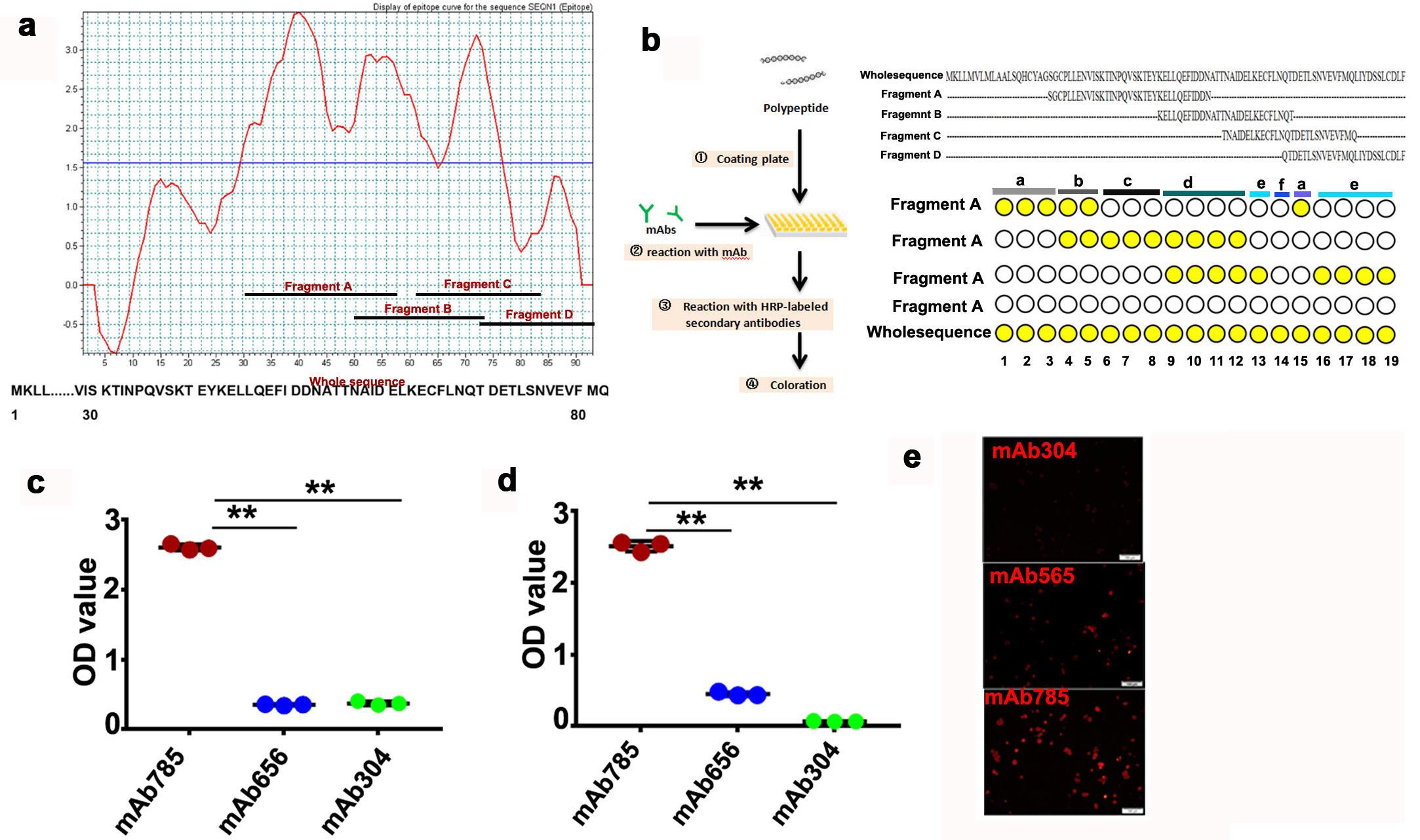


**Figure S1.Epitope prediction and screening of mAbs targeting different epitopes.** a) the epitope curve of Mam-A was mapped and the possible distribution of epitopes within the protein was predicted by BioSun Version 3.0software; b) Based on predicted distribution of potential epitopes, Mam-A was cloned into four polypeptide fragments that may contain different epitopes (Fragment A, B, C and D); ELISA assay revealed the 7 reactive characteristics, representing 7 different epitopes in Mam-A; c) quantifying the reactivities of mAbs (0.5 µg/mL) with the targeting fragment by Elisa; d) quantifying the reactivities of mAbs (0.5 µg/mL) with Mam-A by Elisa. ***P*<0.01; e) immunostained cells by different mAbs targeting different epitopes.

**
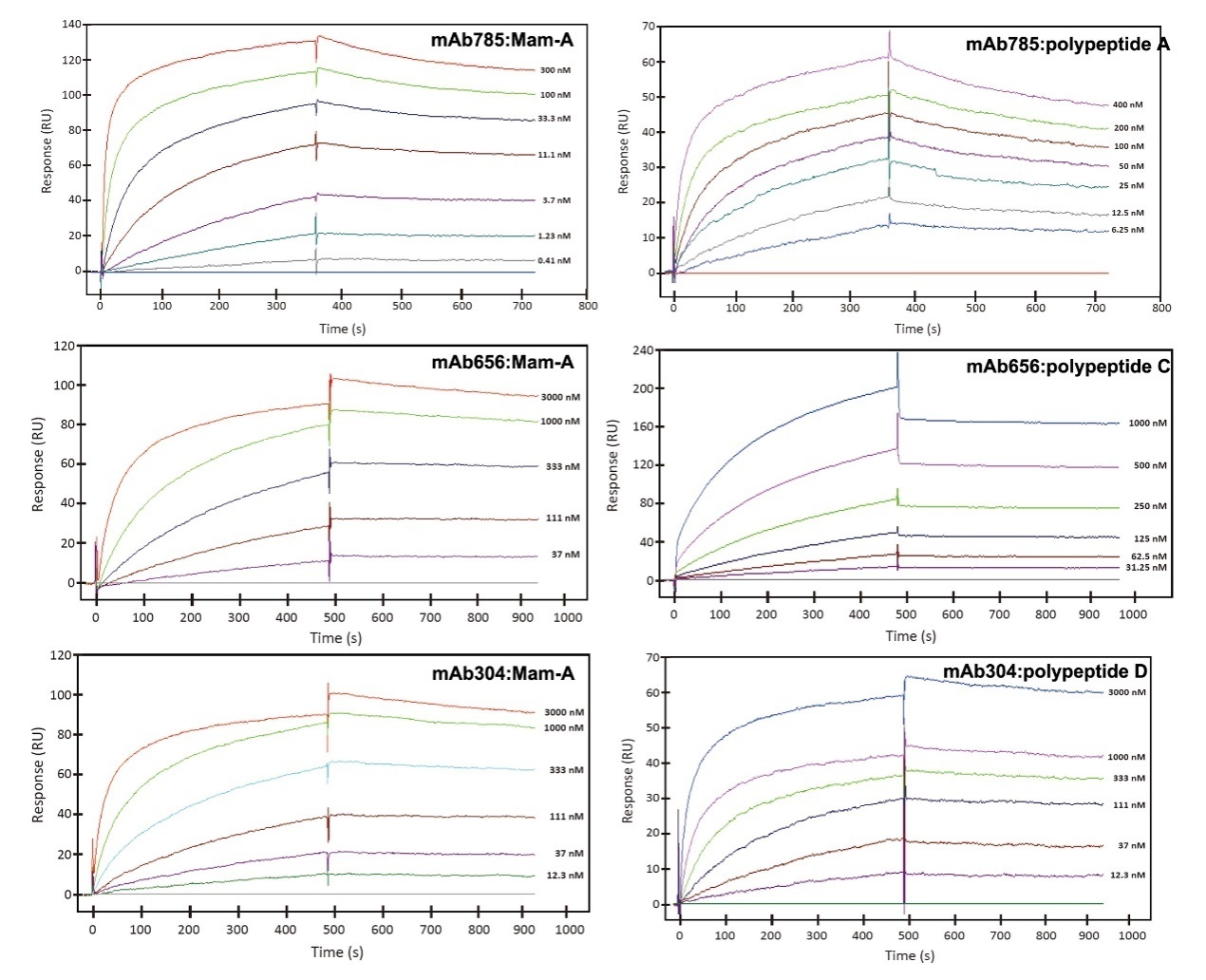
Figure S2. Representative sensorgrams of immobilized mAbs interacting with Mam-A/targeted polypeptides at gradient concentrations.** mAb785 interacting with Mam-A, capturing: 5μL/min (flow rate)×1.2 min (injection time). Interaction: 30μL/min (flow rate), injection time 6 min, dissociation time 6 min. Regeneration: 10mM Glycine-HCl Buffer (pH=1.7), 30μL/min × 30s; mAb785 interacting with polypeptide A, capturing: 5μL/min (flow rate) *1.2 min (injection time). Interaction: 30μL/min (flow rate), injection time 6 min, dissociation time 6 min. Regeneration: 10mM Glycine-HCl Buffer (pH1.7), 30μL/min × 30s; mAb656 interacting with Mam-A, capturing: 5μL/min (flow rate) ×1.2 min (injection time). Interaction: 30μL/min (flow rate), injection time 8 min, dissociation time 8 min. Regeneration: 10mM Glycine-HCl Buffer (pH=1.7), 30μL/min × 30s; mAb656 interacting with polypeptide C, capturing: 5μL/min (flow rate) ×1.2 min (injection time). Interaction: 30μL/min (flow rate), injection time 8 min, dissociation time 8 min. Regeneration: 10mM Glycine-HCl Buffer (pH=1.7), 30μL/min × 30s; mAb304 interacting with Mam-A, capturing: 5μL/min (flow rate) × 1 min (injection time). Interaction: 30μL/min (flow rate), injection time 8 min, dissociation time 8 min. Regeneration: 10mM Glycine-HCl Buffer (pH=1.7), 30μL/min × 30s. mAb304 interacting with polypeptide D, capturing: 5μL/min (flow rate) × 1 min (injection time). Interaction: 30μL/min (flow rate), injection time 8 min, dissociation time 8 min. Regeneration: 10mM Glycine-HCl Buffer (pH=1.7), 30μL/min × 30s.


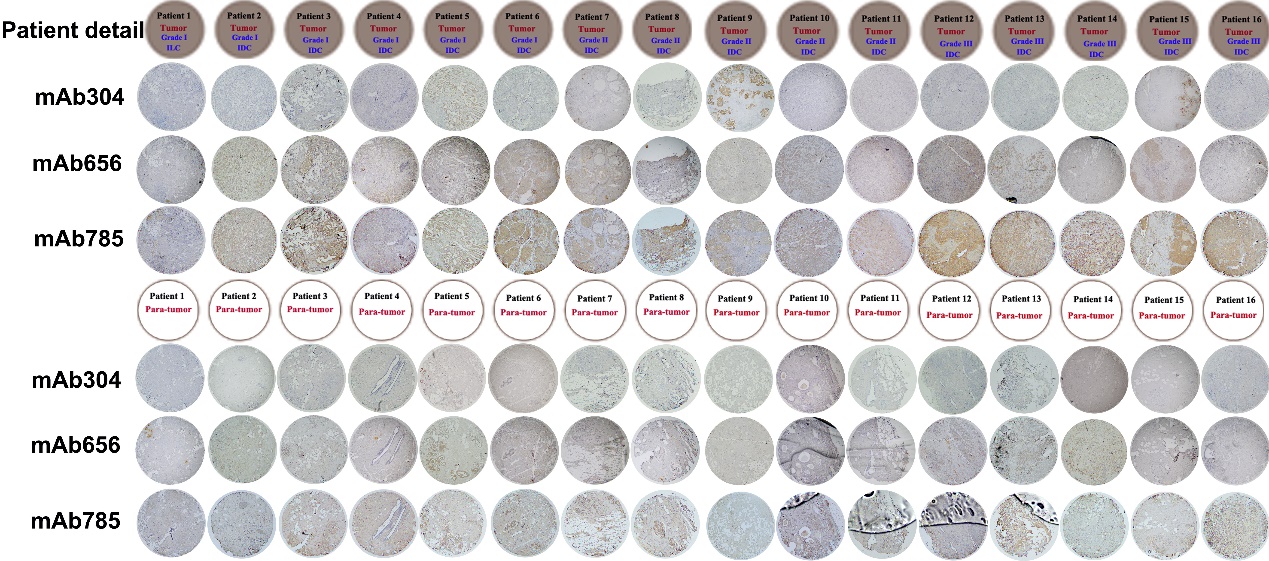


**Figure S3. Immunostaining of clinical breast cancer tissue chips by anti-Mam-A mAbs that target different epitopes.** 16 cases of breast tumors and corresponding paracancerous tissues were included on the chips.


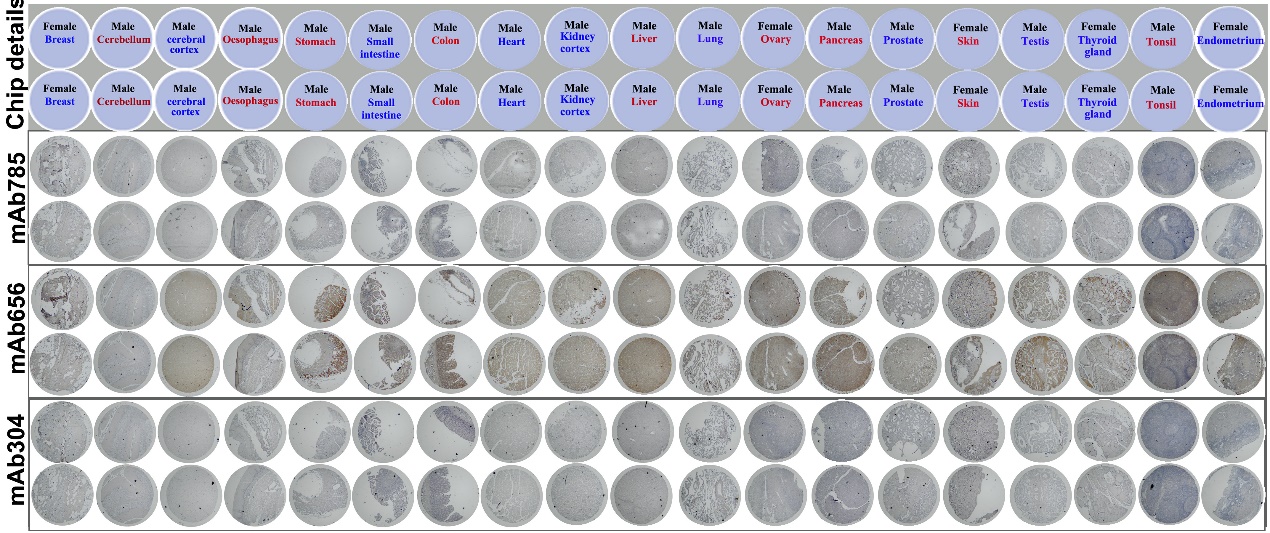


**Figure S4. Immunostaining on normal tissue chips.** Chips including 19 types of normal tissues (two samples for each type) were customized and immunostained by mAb785, mAb656 and mAb304 to evaluate the specificities of mAbs.

**
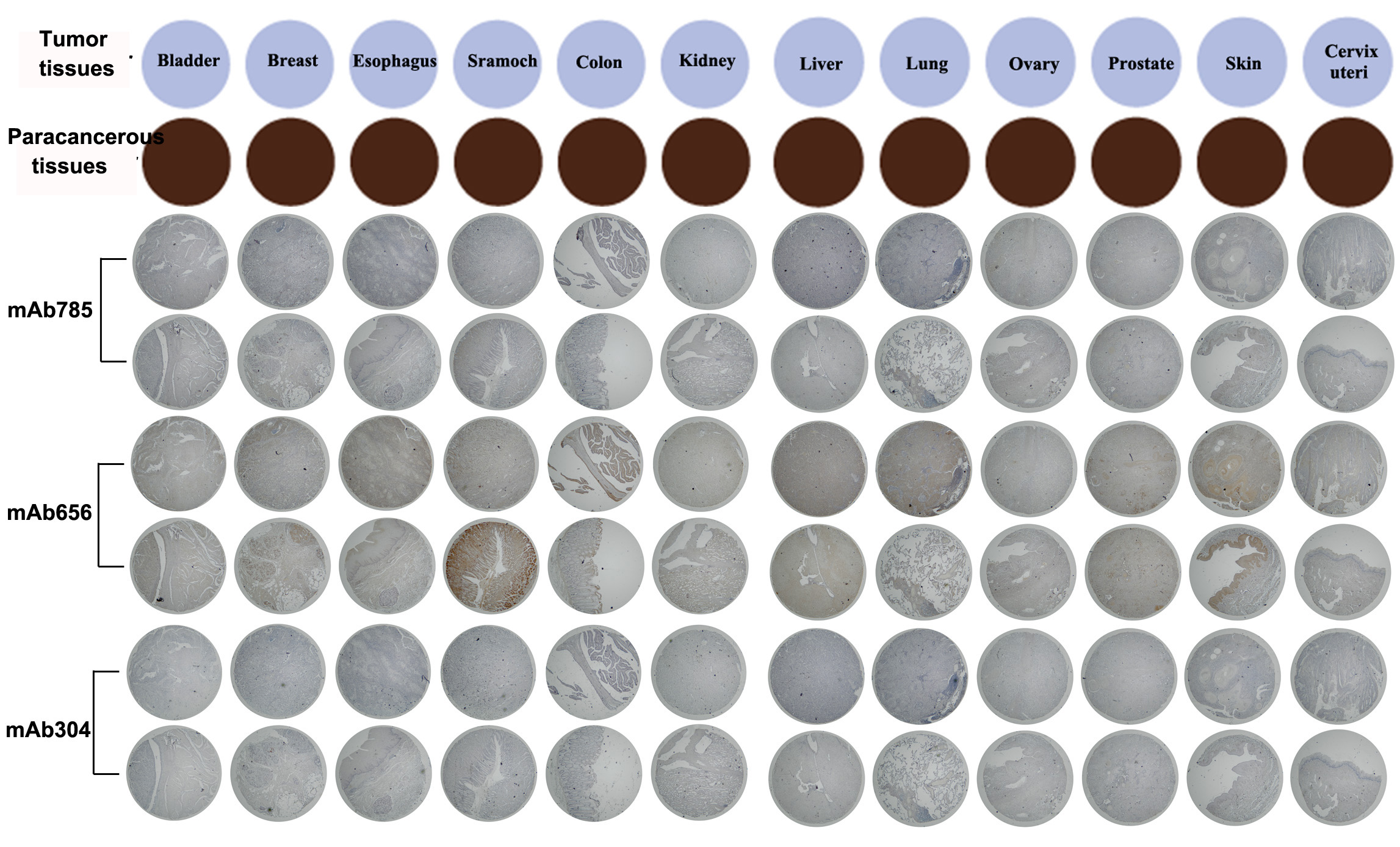
 Figure S5. Immunostaining on non-breast tumor tissue chips.** Chips including 12 types of cancer and paracancerous tissues were customized and immunostained by mAb785, mAb656 and mAb304 to evaluate the specificities of mAbs.


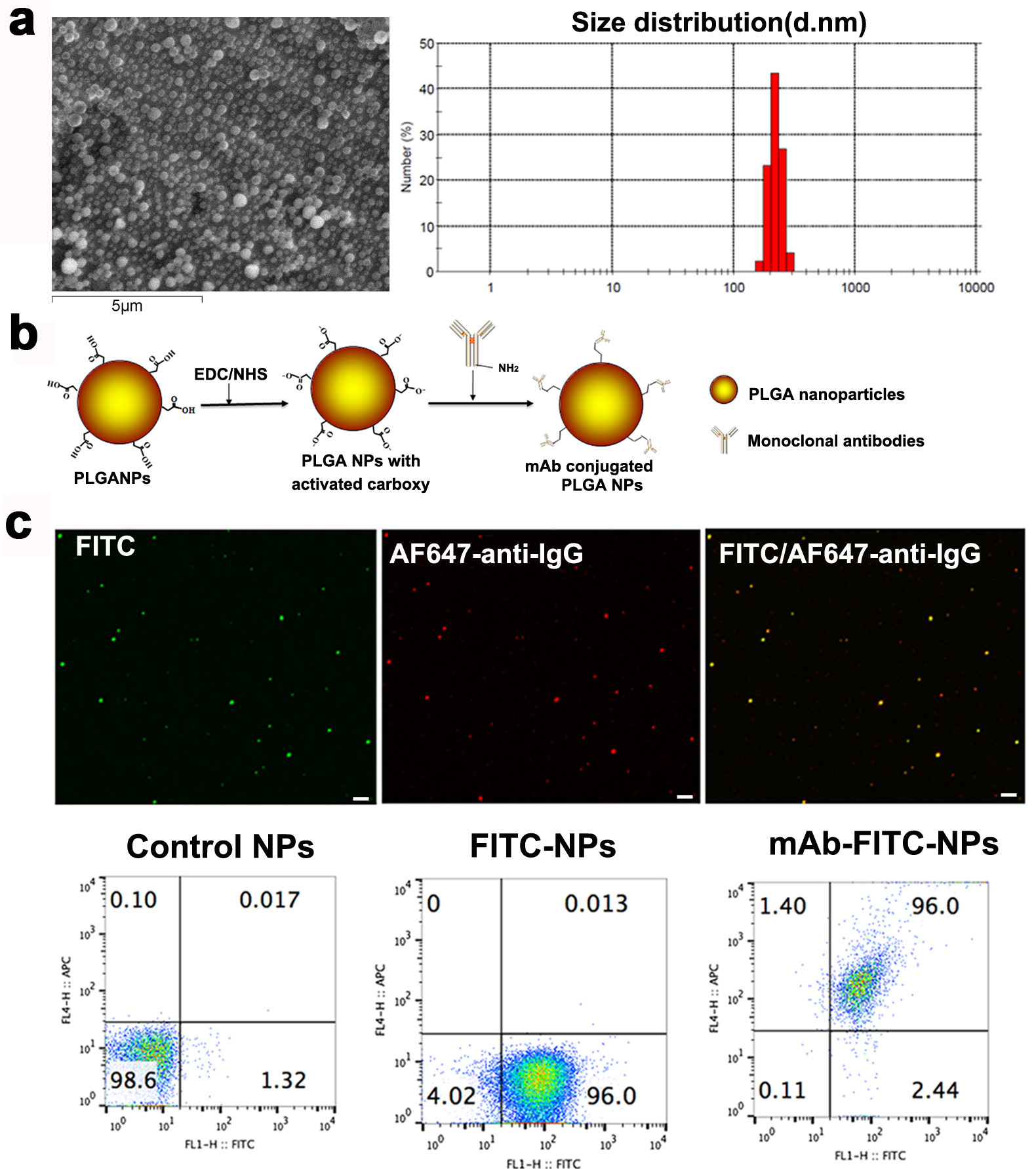


**Figure S6. Preparation of nanoparticles and mAb-conjugated nanoparticles.** a) SEM and size distribution of PLGA nanoparticles; b) schematic illustrating the conjugation of mAbs to nanoparticles; c) FITC dyes were incorporated into NPs for labeling. After blocking with BSA, NPs were immunostained with AF647-labeled anti-mouse IgG to determine the conjugation of mAbs on the NPs. Co-localization of AF647 and FITC indicated the successful conjugation of mAb on NPs. Flow cytometry provided further evidence for the successful conjugation of mAb on NPs and demonstrated that more than 95% NPs were successfully conjugated.


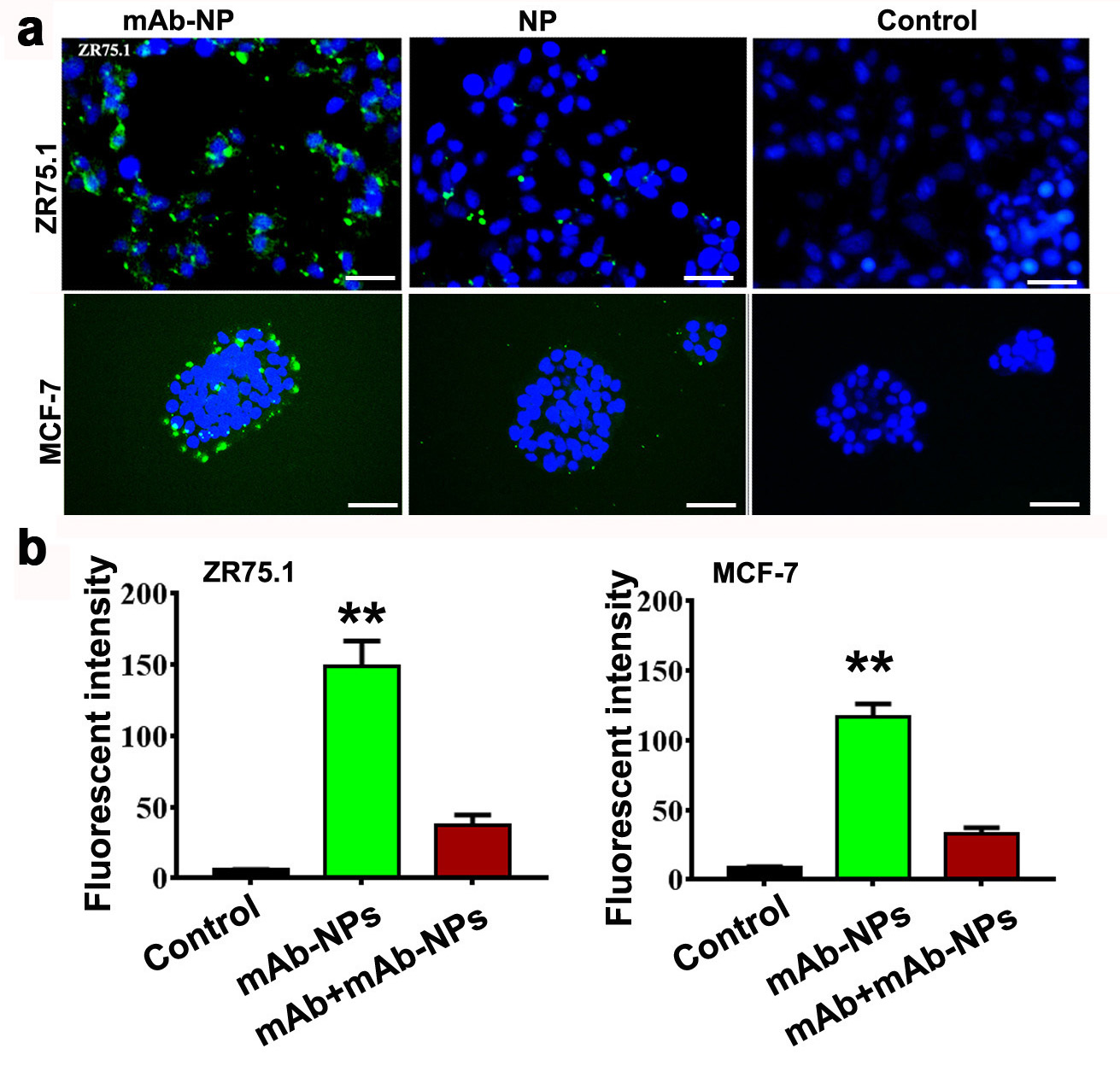


**Figure S7. Cellular uptake of FITC-incorporated NPs by breast cancer cells.** a) fluorescent microscopy of nanoparticles binding with breast cancer cells, two breast cancer cell lines ZR75.1 and MCF-7 were used; b) Quantified fluorescent intensity of FITC from cells treated by mAb785-NPs or plain NPs (***P*<0.01).


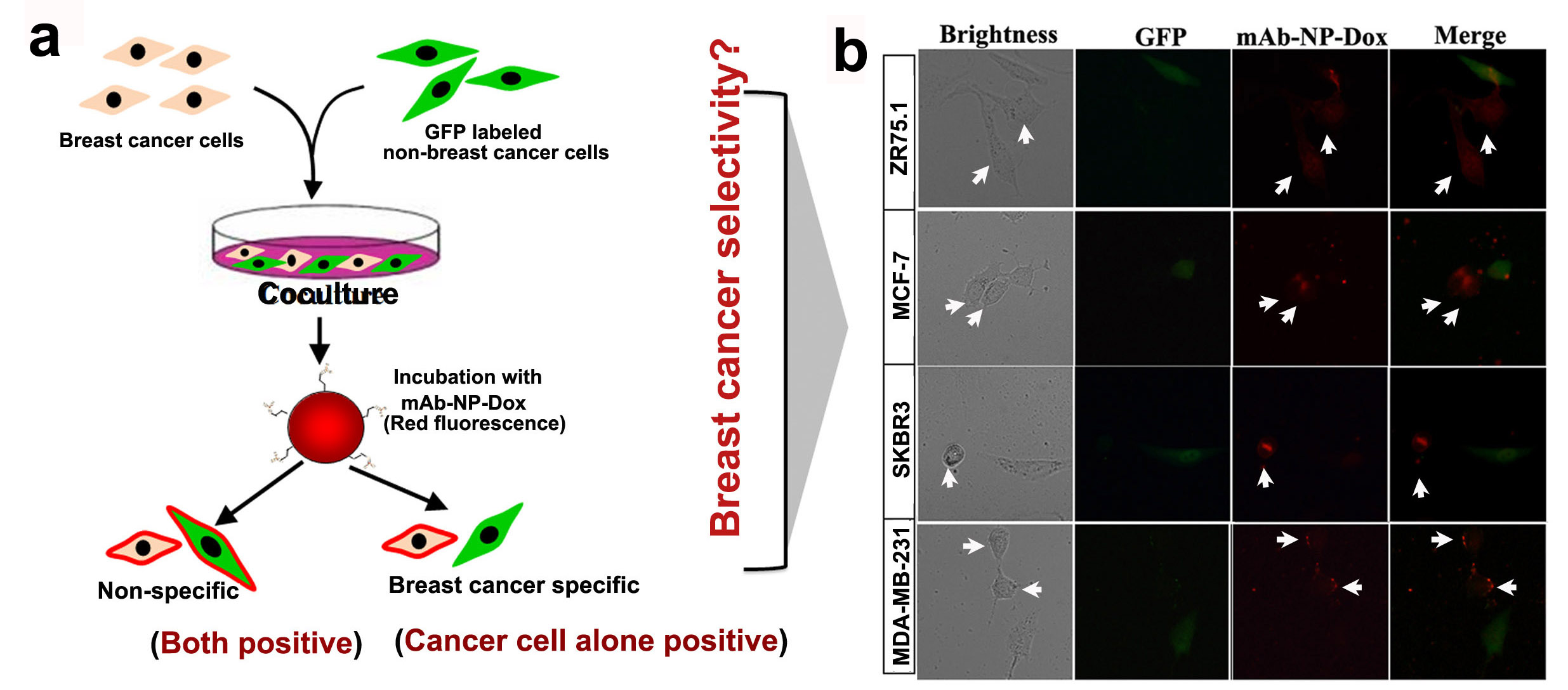


**Figure S8. Specific targeting of mAb785-conjugated nanoparticles to breast cancer cells.** a) The schematic specificity assay of mAb785-NPs(Loading Dox, red fluorescence) to breast cancer cells . b) The specific tatgeting of mAb785-NPs to breast cancer cells confirmed through fluorescent distribution in breast cancer cells VS co-cultured with non-breast cancer cells.


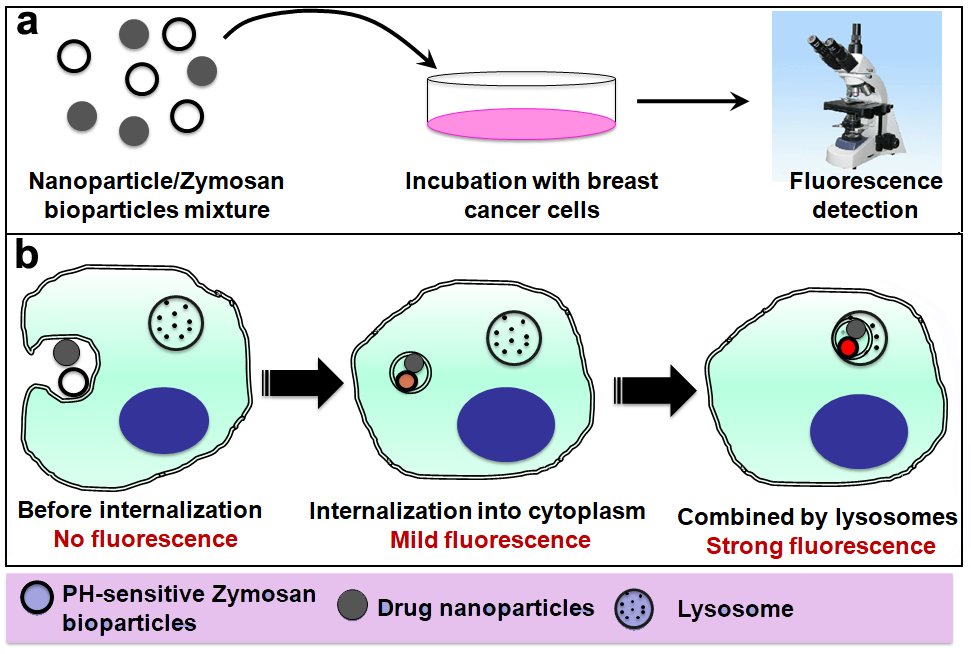


**Figure S9. Schematic illustration of nanoparticle phagocytosis by cells.** a) Drug nanoparticles were mixed with pHrodo^TM^ Red conjugated Zymosan bioparticles (a phagocytosis indicator) and then, the mixed particles were incubated with breast cancer cells. Fluorescent signals were observed to monitor the internalization process of particles; b) The principle of Zymosan bioparticle indicating the internalization process of drug nanoparticles.


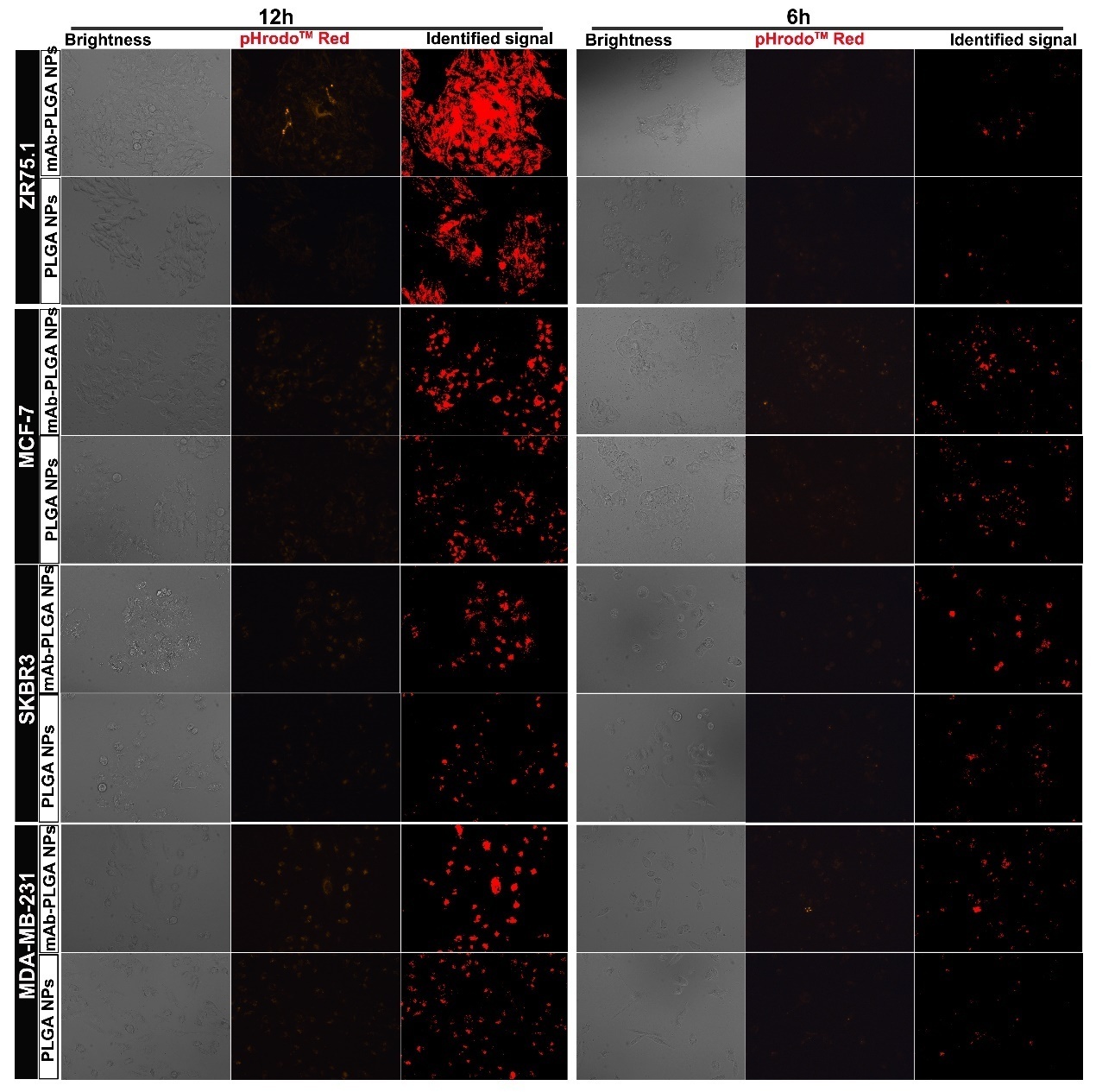


**Figure S10. mAb785-epitope interaction promotes the uptake of nanoparticles by multiple breast cancer cells.** Phagocytosis indicator pHrodoTM Red conjugated Zymosan Bioparticles were mixed with mAb785-conjugated or control nanoparticles. The mixed particles were incubated with four genotypes of breast cancer cells respectively, and the uptake of nanoparticles was observed by the indicator fluorescence at 6 and 12h.


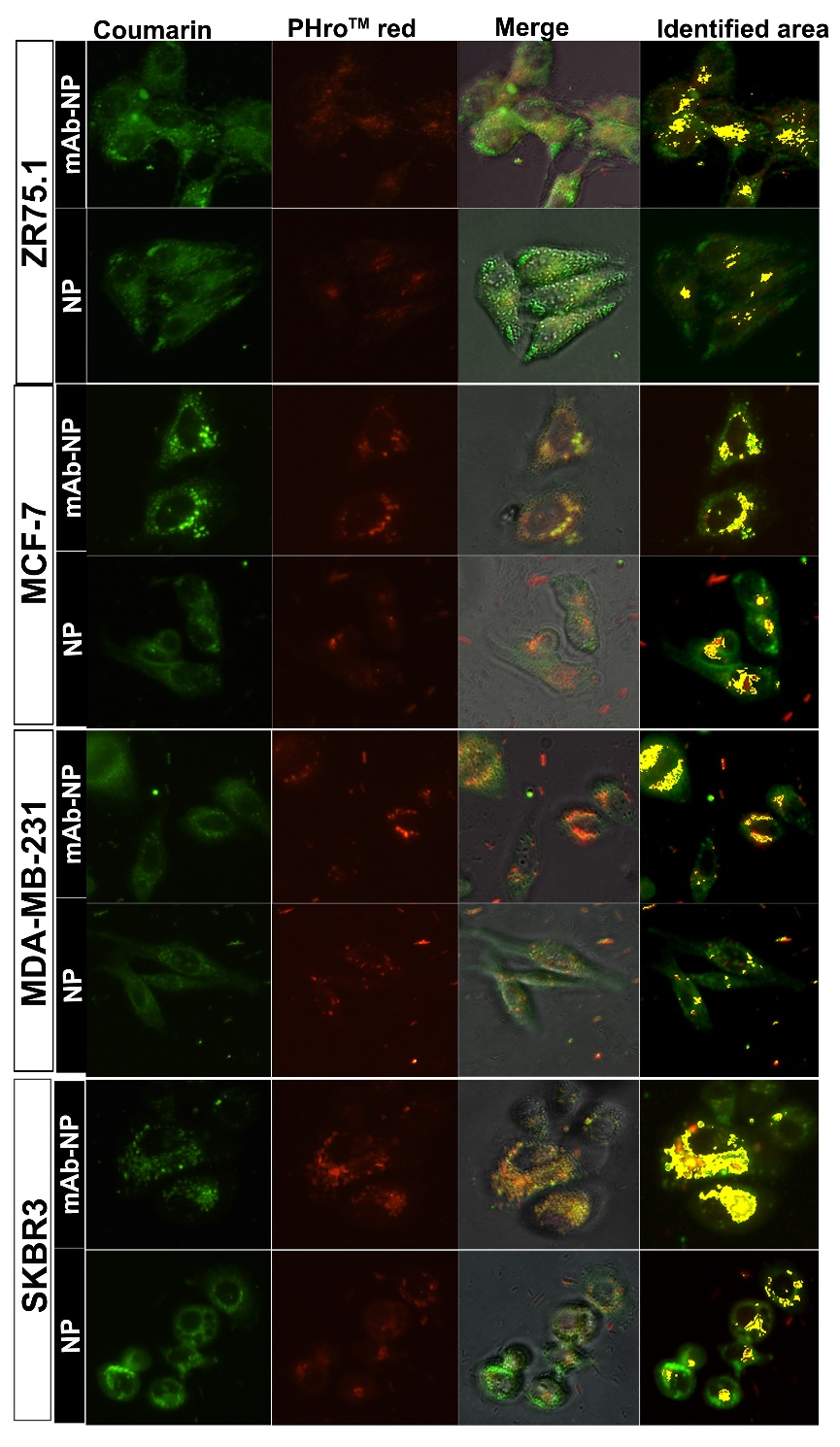


**FigureS11. Uptake of nanoparticles by multiple breast cancer cells.** Coumarin-labeled (Green) nanoparticles were mixed with phagocytosis indicator pHrodoTM Red, and the uptake of nanoparticle was determined as co-localization of green and red fluorescence.


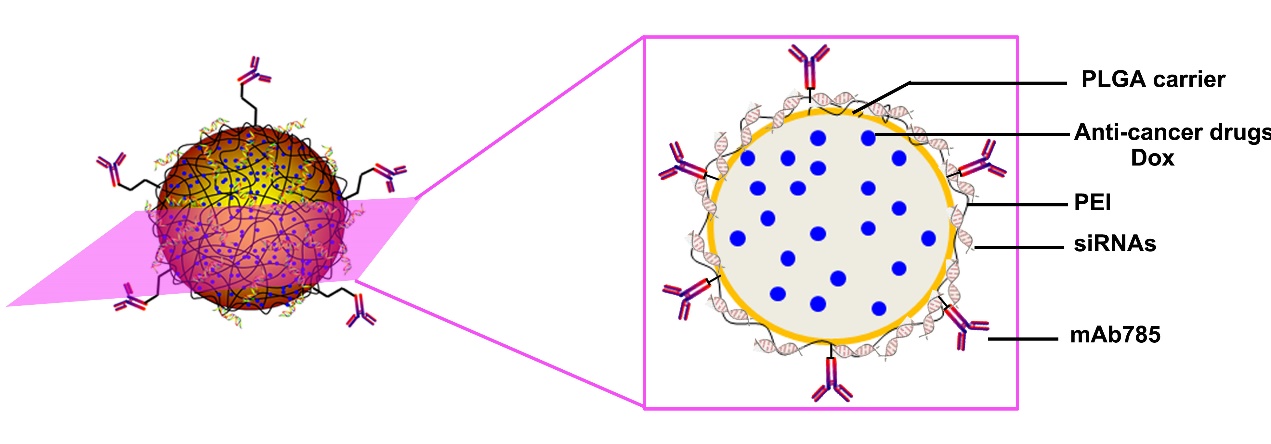


**Figure S12. Schematic representation for design and structure of Nanodrugs.**


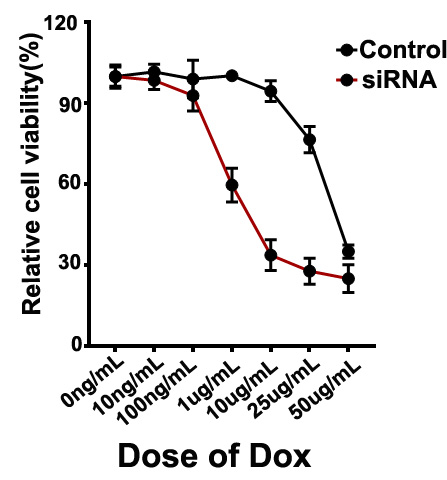


**Figure S13.The effect of survivin gene silencing on the sensitivity of breast cancer cells to Dox.** SiRNA indicated cells whose survivin genes were silenced by RNA interference. Dox was incubated with cells for 24h. The data suggested that silencing survivin gene by siRNAs significantly enhanced the sensitivity of breast cancer cells to Dox.


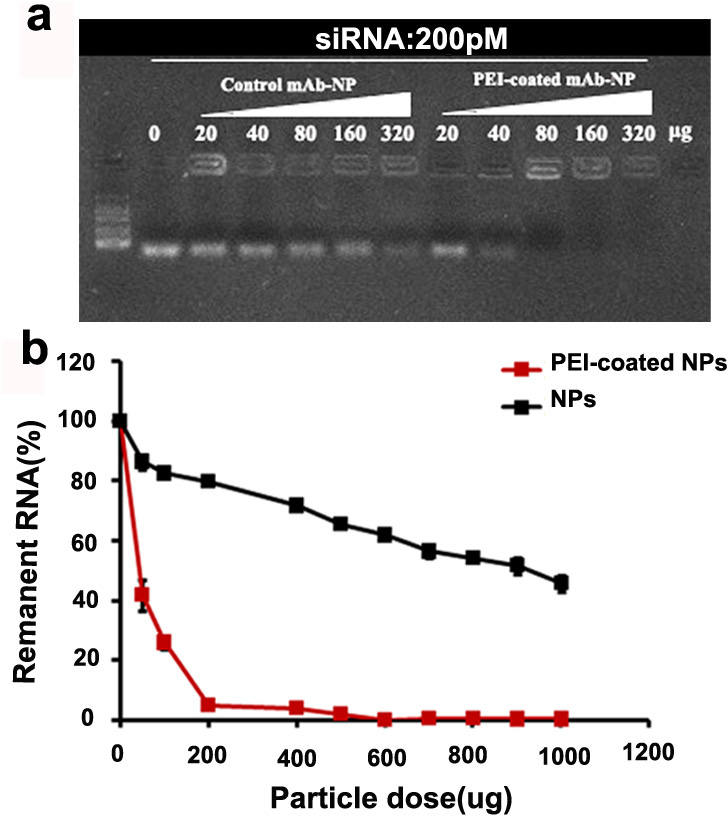


**Figure S14. Comparison of PLGA NPs and PEI-coated PLGA NPs in loading siRNAs.** a) 500pmol siRNAs were loaded onto normal NPs or PEI-NPs of different doses in distilled water. A gel retardation assay was used to identify NPs completely combined with siRNAsat the different ratio of NPs: siRNAs. b) The NPs were removed by centrifugation and the unloaded siRNAs in the supernatant were measured using spectrophotometer;


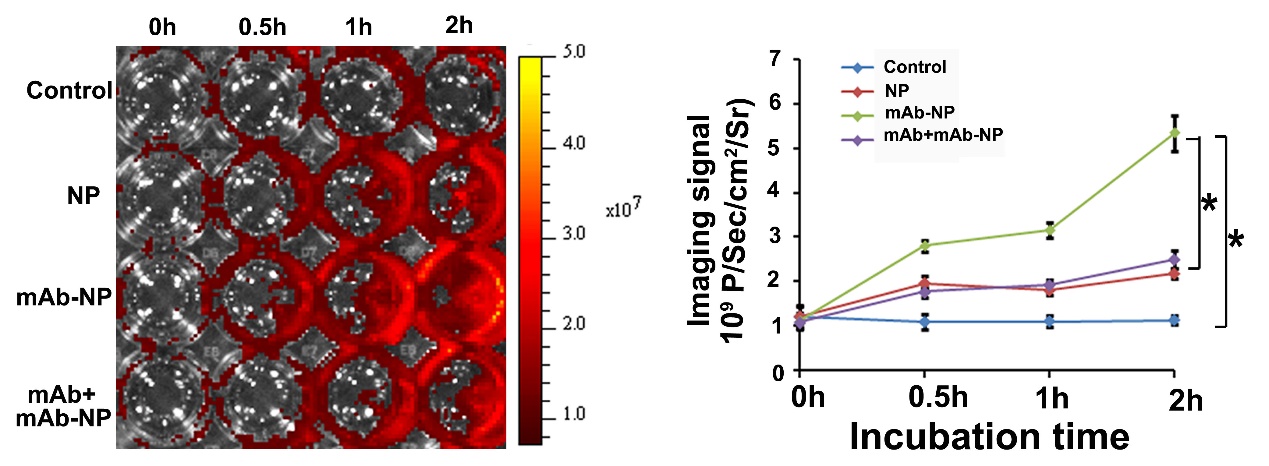


**Figure S15.Targeted binding of nanoparticles carrying Cy5-siRNA to breast cancer cells.** The optical imaging at near infrared region was performed to detect bond nanoparticles (mAb+mAb-NPs meant that the cells were pre-incubated with mAbs for 2h and then incubated with mAb-NPs; Quantitative analysis of imaging signals was performed)(**P*<0.01);


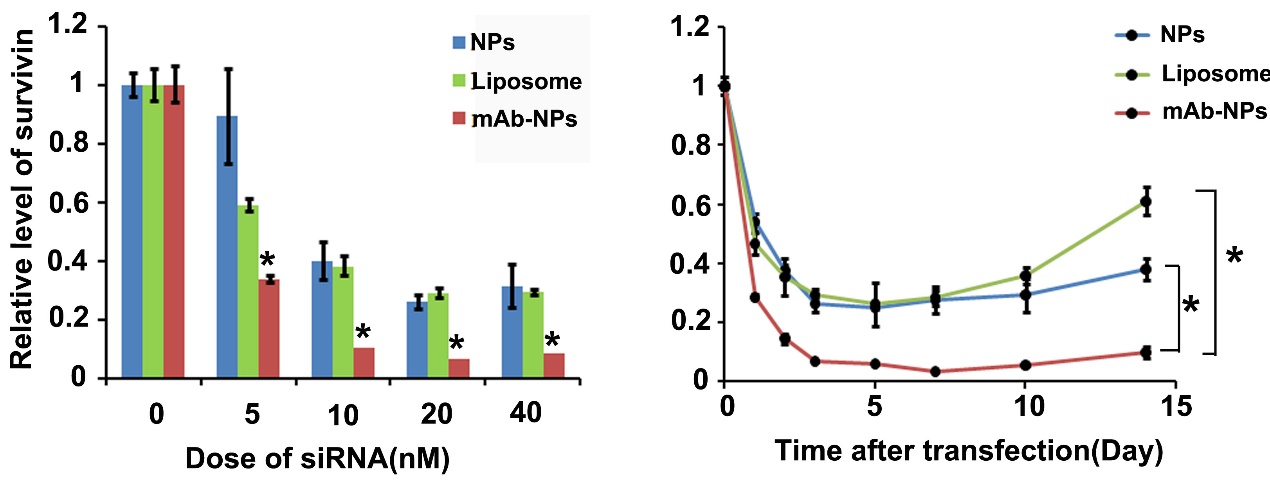


**Figure S16. Dose-and time-dependent survivin gene silencing in breast cancer cells mediated by liposomes, NPs and mAb785-NPs.** **P*<0.01 compared with the other groups.


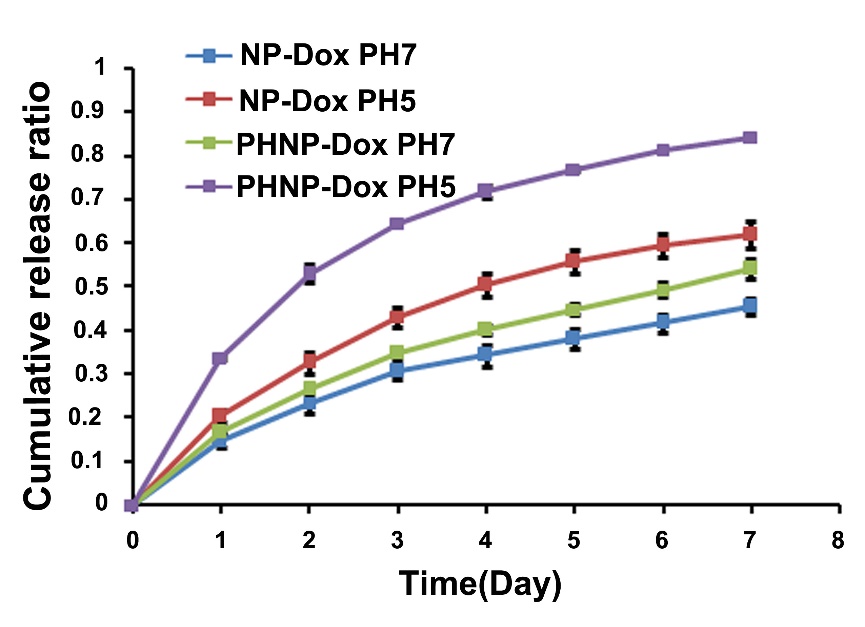


**Figure S17. The release profiles of Dox from PLGA NPs and pH-responsive PLGA NPs (PHNPs) *in vitro*.**

**
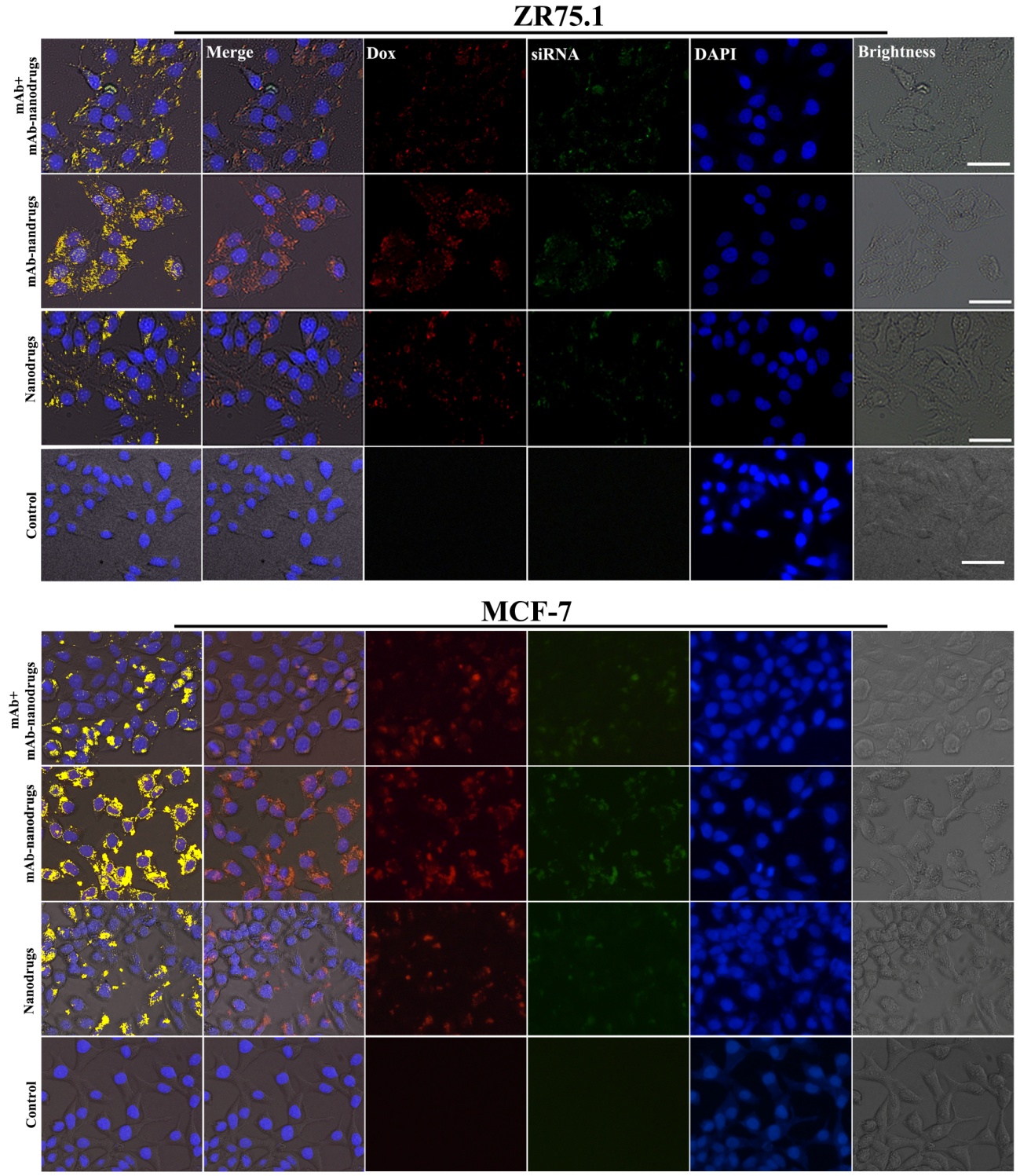
Figure S18.The targeted delivery of Nanodrugs (carrying Dox and FAM-siRNA) into ZR75.1 and MCF-7 cells.**(Bar=50μm).

**
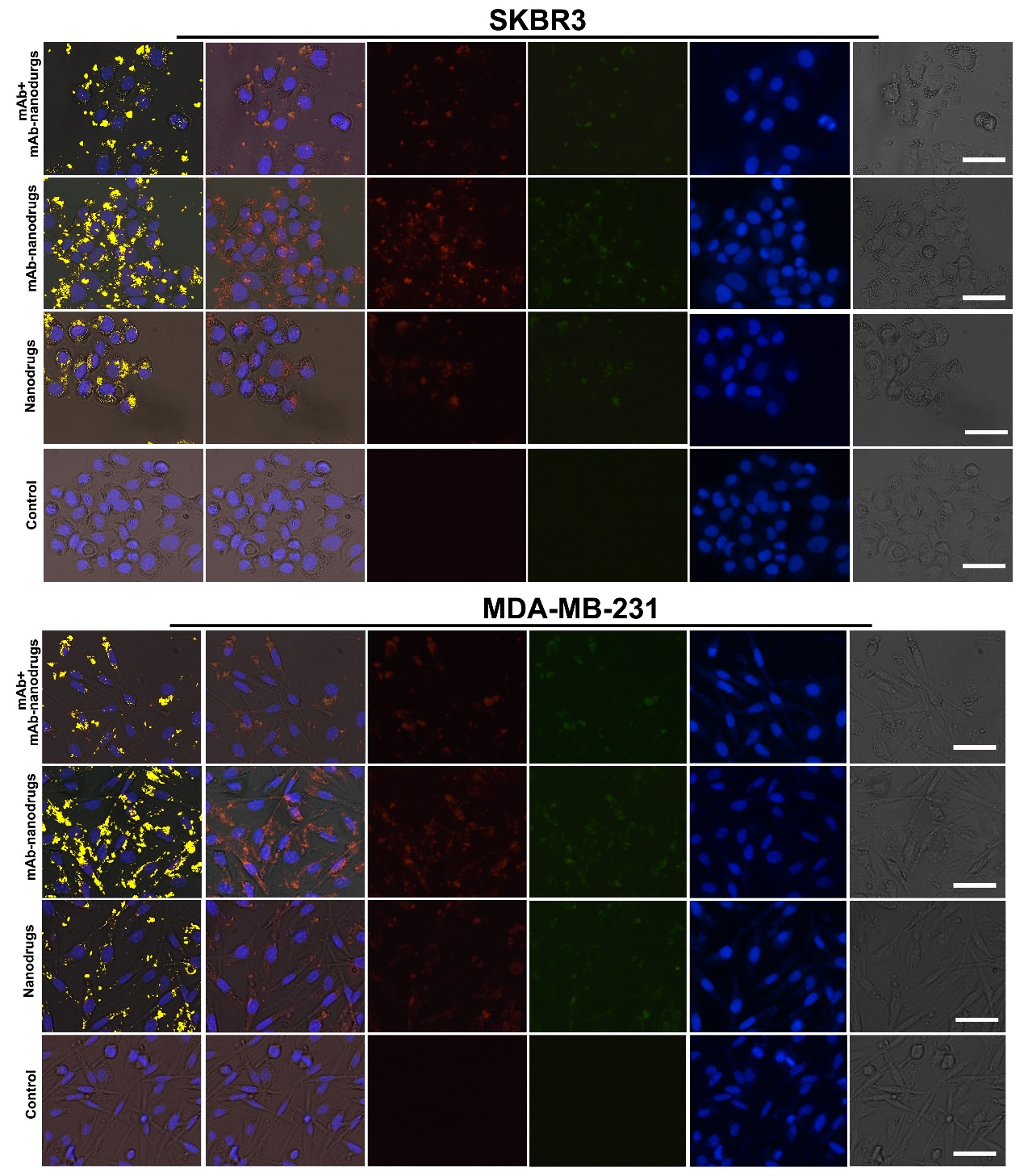
**

**Figure S19. The targeted delivery of Nanodrugs(carrying Dox and FAM-siRNA) into SKBR3 and MDA-MB-231 cells**(Bar=50μm).


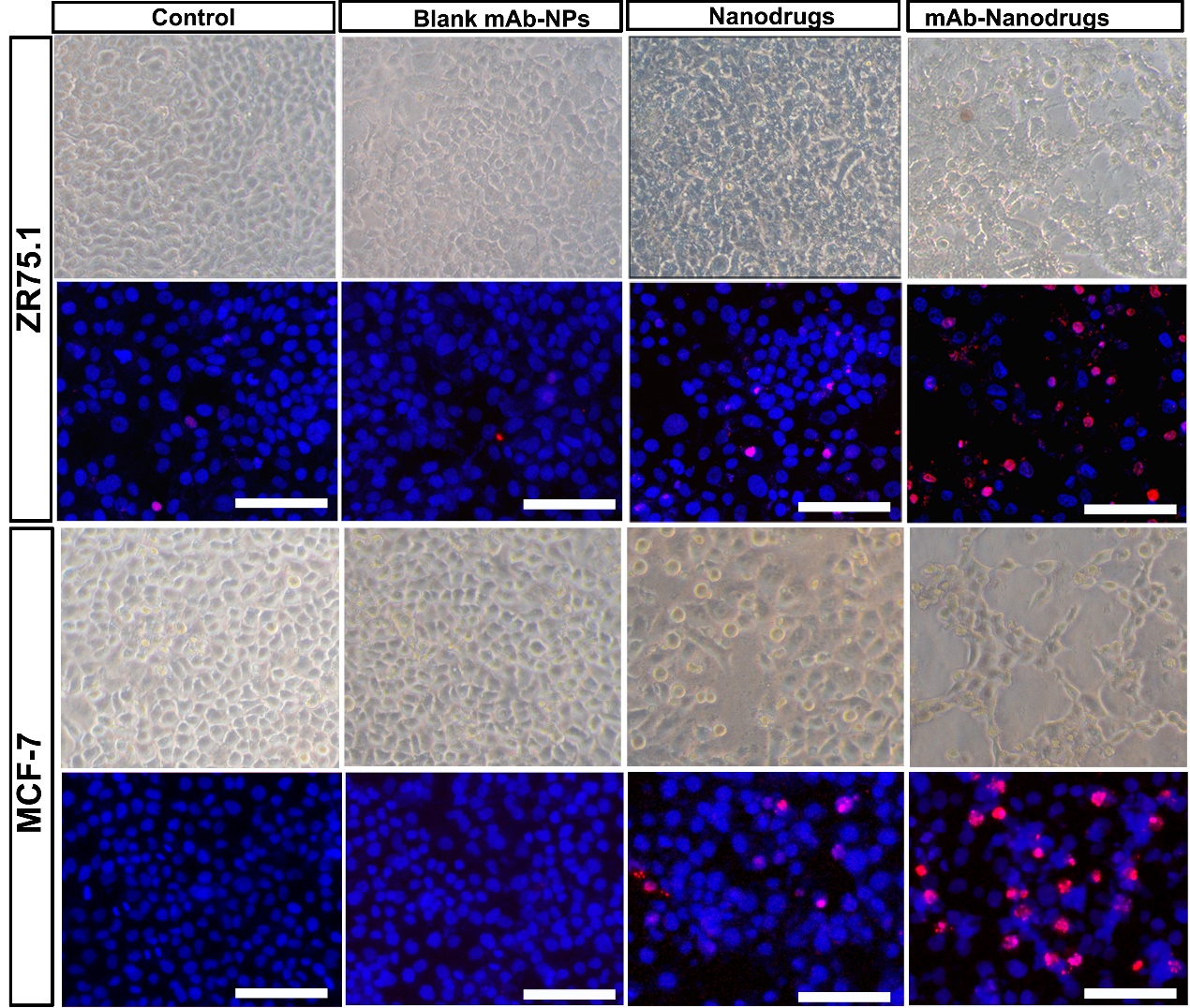


**Figure S20.The targeted inhibition of multiple breast cancer cells *in vitro* by mAb-Nanodrugs (carrying Dox and FAM-siRNA)**.


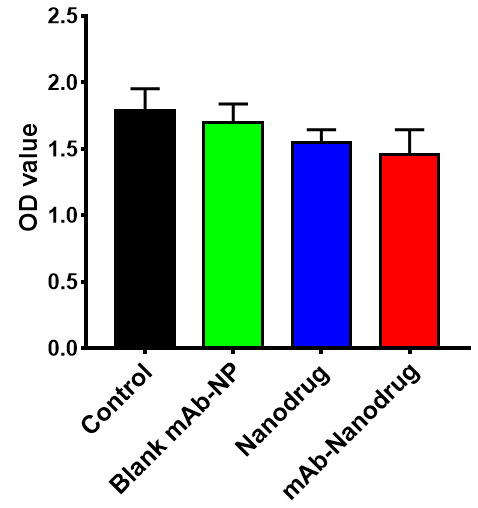


**Figure S21.Evaluating the response of non-breast cancer cells to the targeting nanodrug.** Irrelevant cancer cell A549 was incubated with blank mAb-NP, control Nanodrug or mAb785-Nanodrug respectively for 2h. Redundant Nanodrug was removed by replacing the medium with fresh one. Cells were cultured for additional 2 days and used for CCK8 assay (n=3).


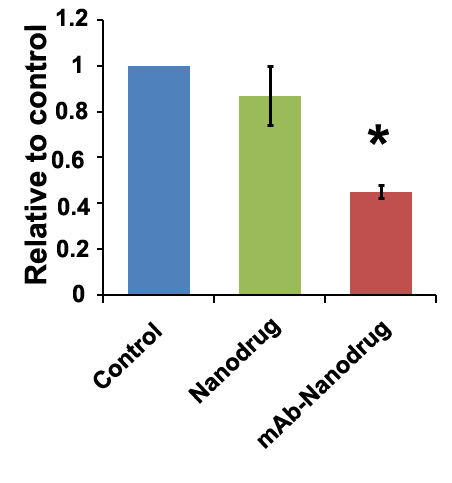


**Figure S22.The targeted inhibition of survivin genes in breast cancers *in vivo* by mAb-Nanodrugs (**P*<0.01 Compared with other group)**.


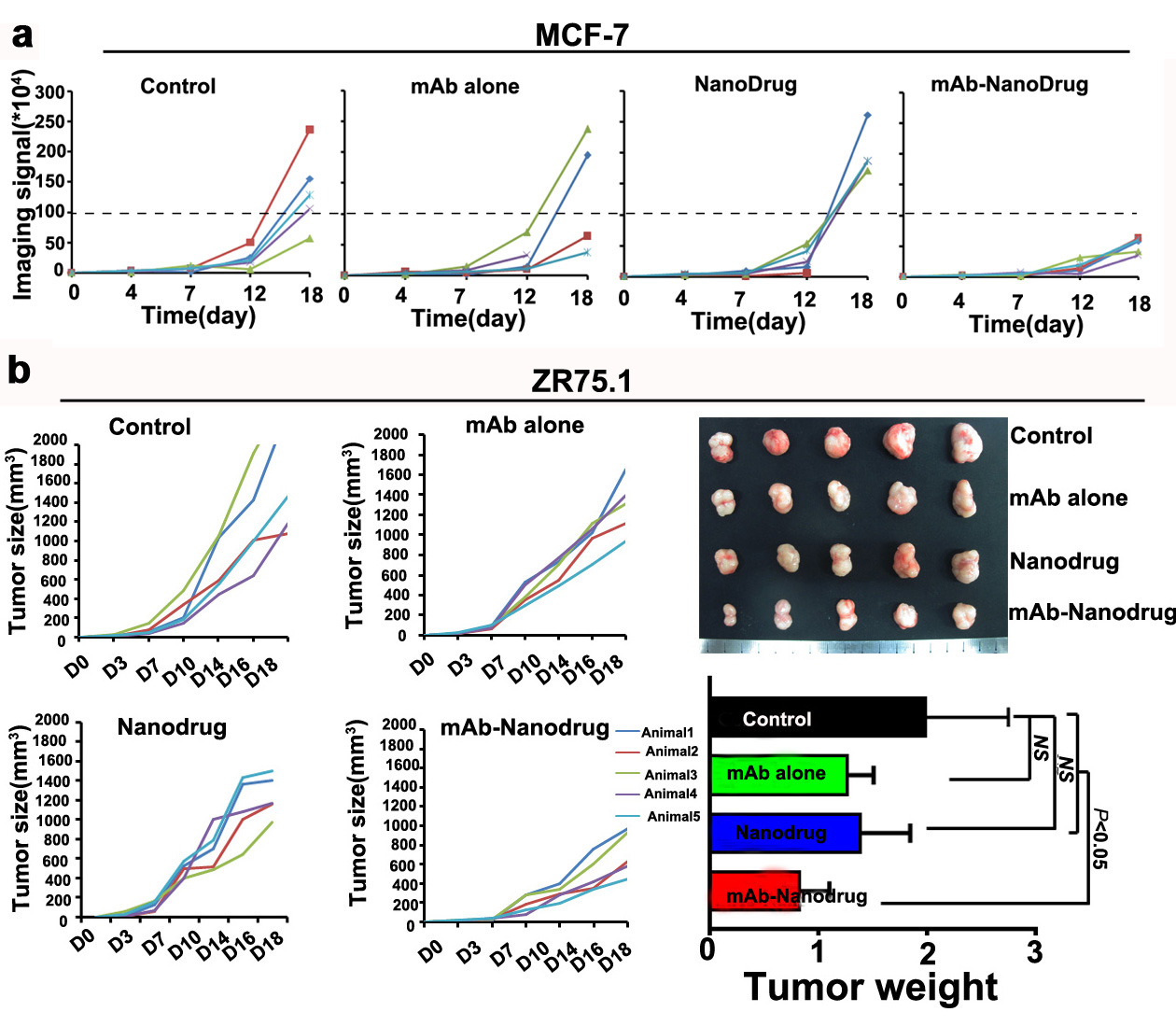


**Figure S23. Inhibition and MCF-7 tumors and ZR75.1 tumors *in vivo* by mAb-Nanodrugs** a) Bioluminescent imaging signals of MCF-7 tumors in each mice receiving different treatment; b) Inhibition of ZR75.1 breast cancer *in vivo* by mAb-Nanodrugs.


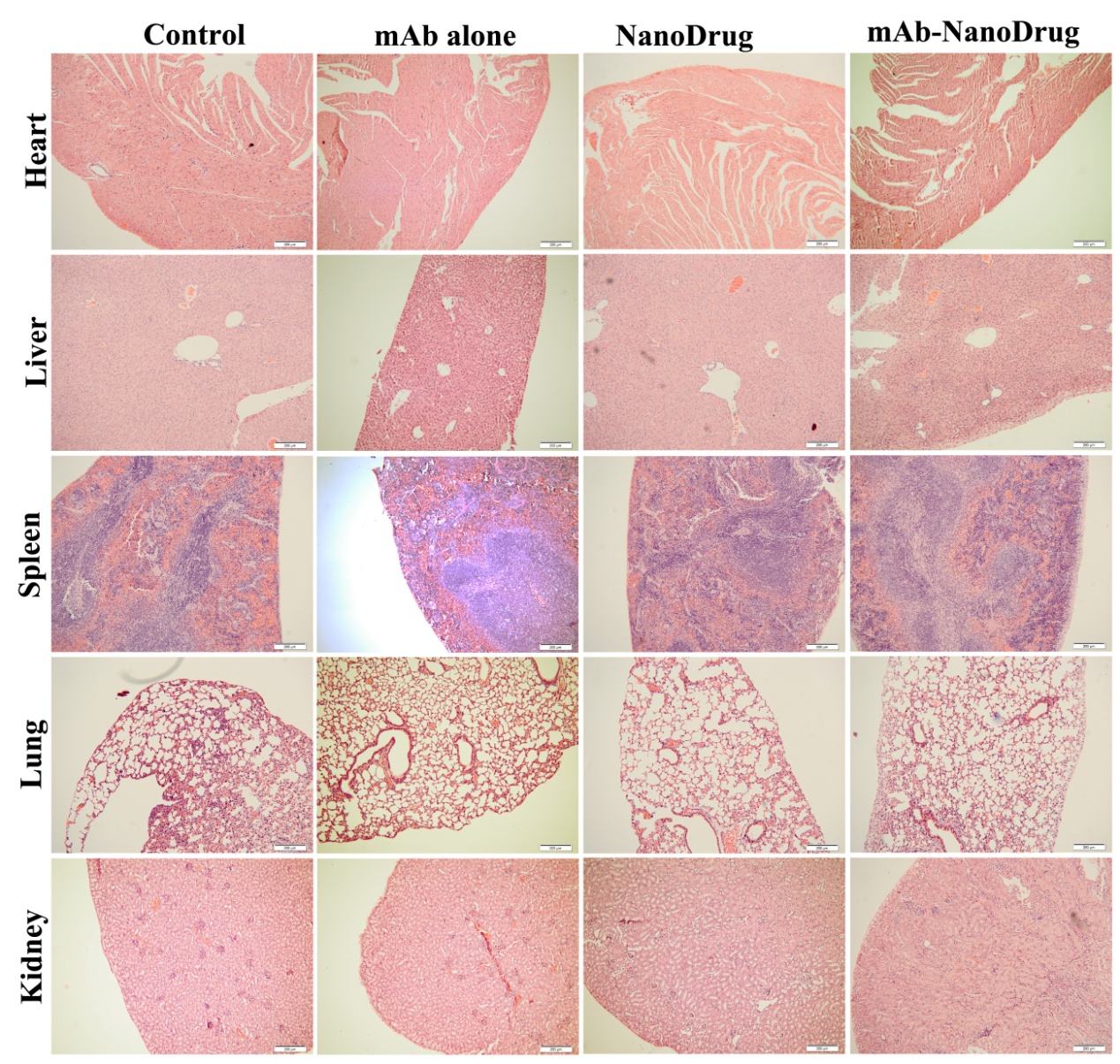


**Figure S24. Safety evaluation of different formulations.** No significant side effect was observed in Nanodrug and mAb-Nanodrug-treated animals.


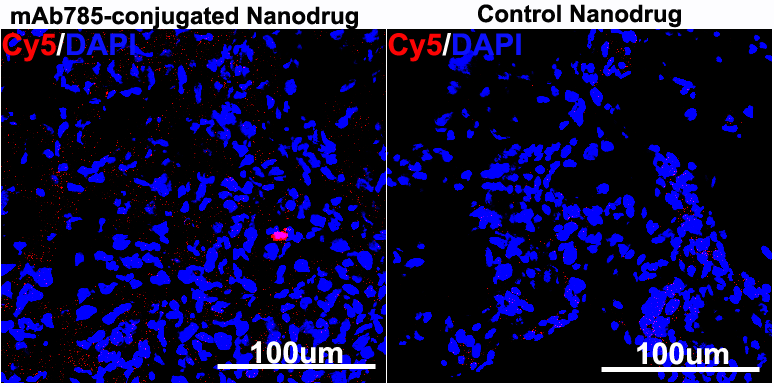


**Figure S25. In vivo targeting of mAb785-conjugated Nanodrug to TNBC tumors.** Nanodrugs were labeled with Cy5 (Loading cy5-siRNA) and tumor sections were detected with fluorescent microscope.


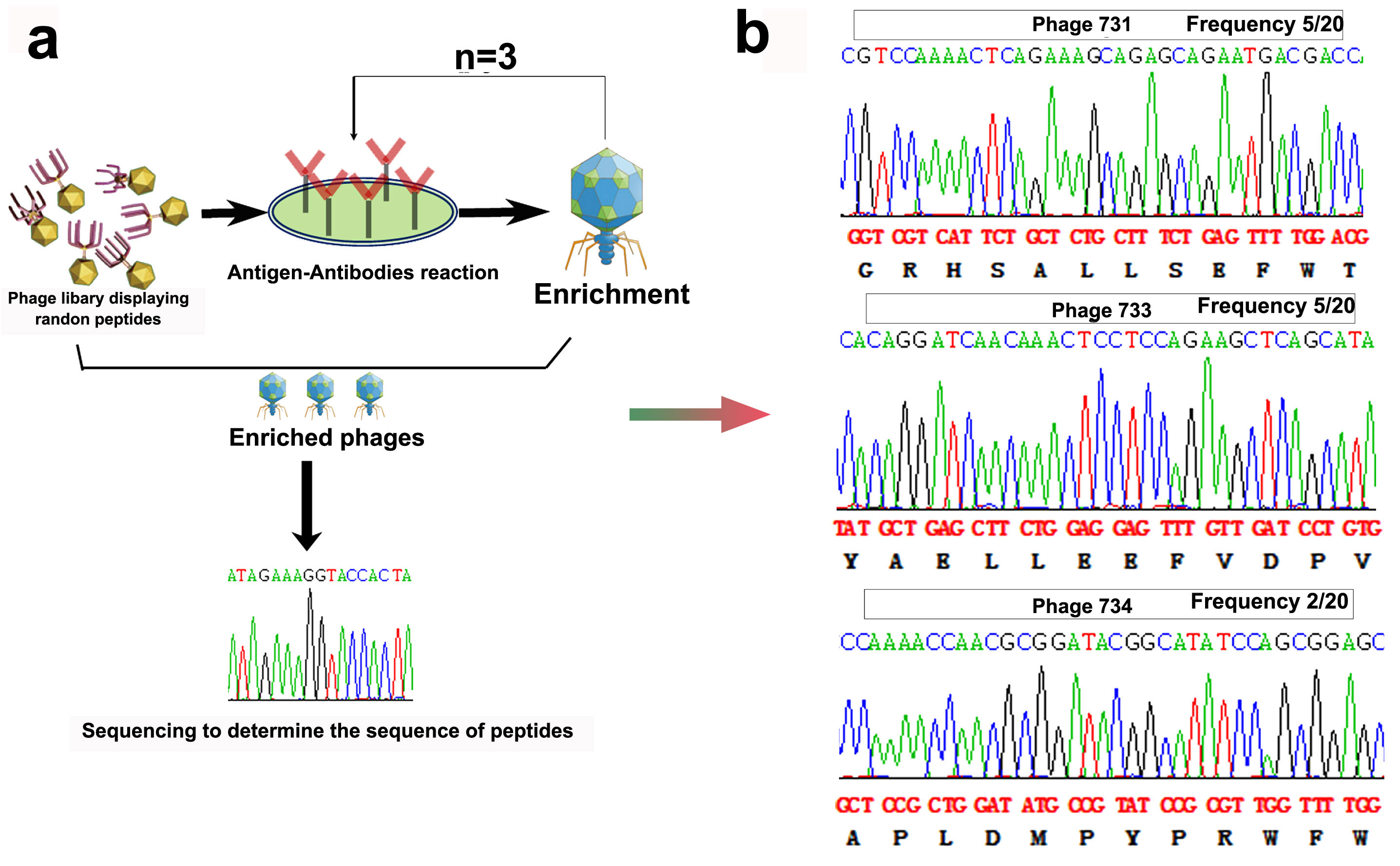


**Figure S26. Phage display screening the potential peptides that were targeted by mAb785.** a) schematic illustrating the screening process by phage display technology; b) Sequencing of the enriched phages determined three high-frequently occurring peptides.

**
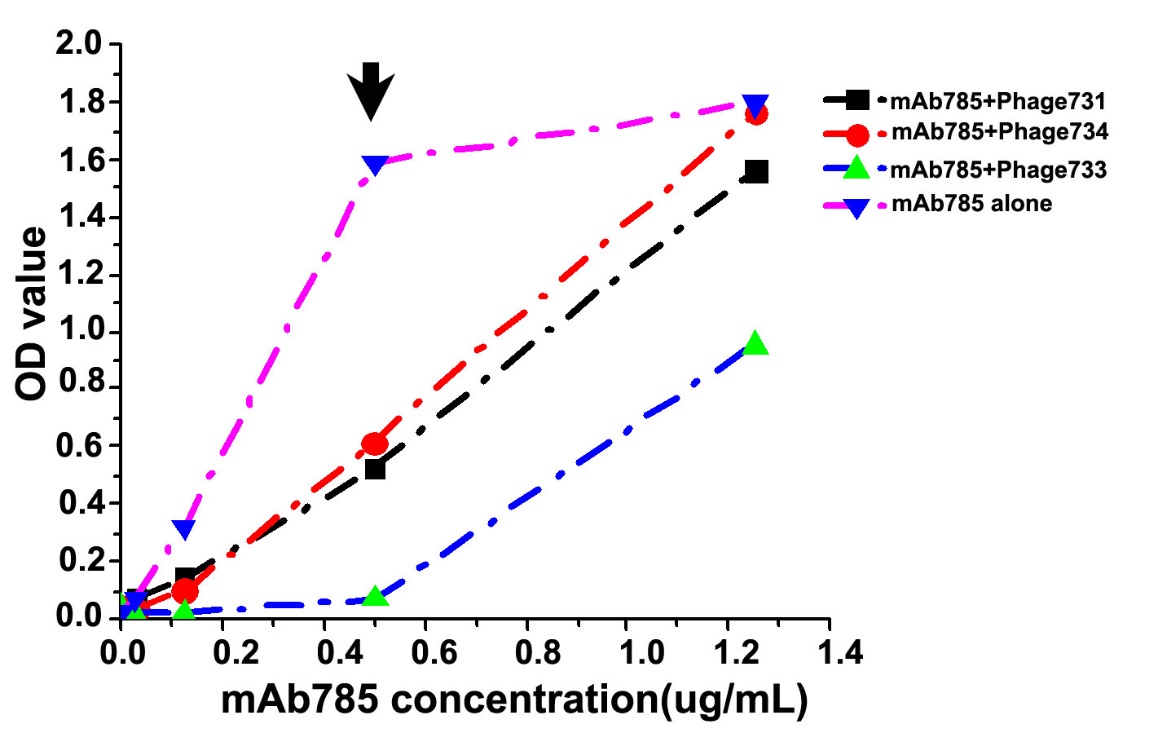
**

**Figure S27. ELISA assaying the blocking efficacies of mAb785-Mam-A reaction by three phages.**


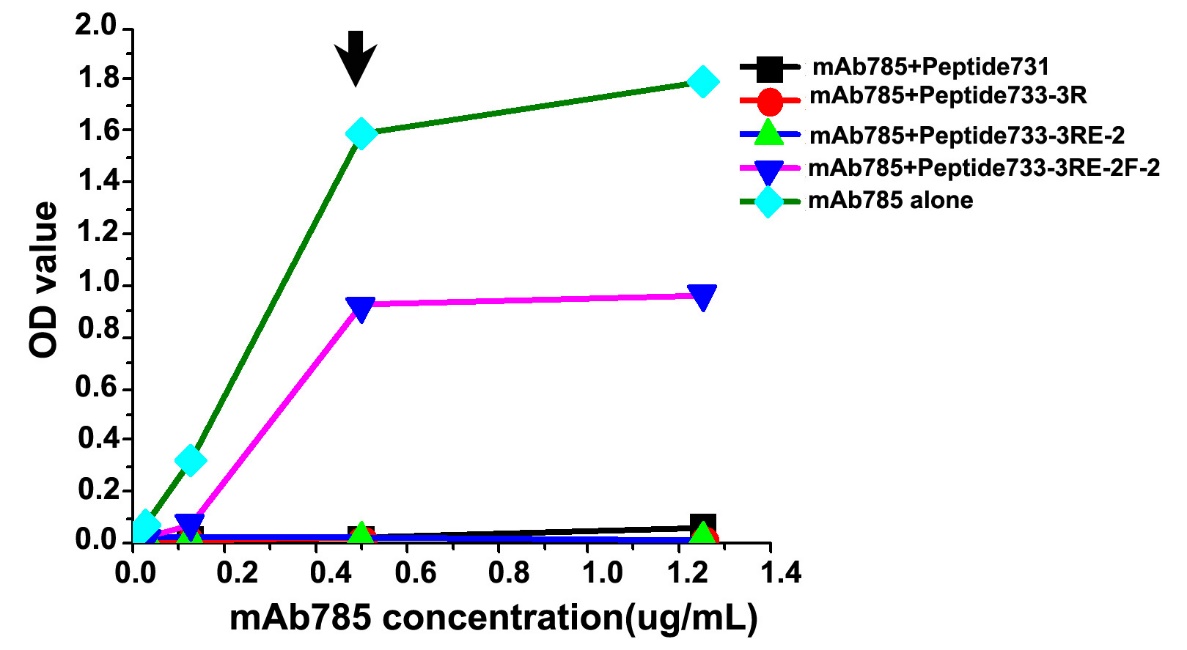


**Figure S28. Elisa assaying the blocking efficacies of mAb785-Mam-A reaction by different peptides.**

**Antibody sequences of mAb785**

**Heavy chain: DNA sequence(396bp)**

Signal sequence-FR1-CDR1-FR2-CDR2-FR3-CDR3-FR4

ATGAACTTCGGGGTCAGCTTGATTTTCCTTGTCCTTGTTTTAAAAGGTGTCCAGTGTGAAGTGAAACTAGTGGAGTCTGGGGGAGGCTTAGTGAAGCCTGGAGGGTCCCTGAAACTCTCCTGTGCAGCCTCAGGATTCACTCTCAGTAACCATGGCATGTCTTGGGTTCGCCAGACTTCAGAGAAGAGGCTGGAGTGGGTCGCATCCATTAGTATTCGTGGTAACACCTACTATCTAGACAGTGTGGAGGGCCGATTCACCATCTCCAGAGATGATGCCAGGAACATCCTGTATCTTCAAATGAGCGGTCTGAGGTCTGAGGACACGGCCATATATTACTGTACATATTTTACTTCGGTAGTCTGGGGCCAAGGCACCACTCTCACAGTCTCCTCA

**Heavy chain: Amino acid sequence (132aa)**

Signal peptide-FR1-CDR1-FR2-CDR2-FR3-CDR3-FR4

MNFGVSLIFLVLVLKGVQCEVKLVESGGGLVKPGGSLKLSCAASGFTLSNHGMSWVRQTSEKRLEWVASISIRGNTYYLDSVEGRFTISRDDARNILYLQMSGLRSEDTAIYYCTYFTSVVWGQGTTLTVSS

**Light chain: DNA sequence (393bp)**

Signal sequence-FR1-CDR1-FR2-CDR2-FR3-CDR3-FR4

ATGAGTCCTGCCCAGTTCCTGTTTCTGTTAGTGCTCTGGATTCGGGAAACCAACGGTGATGTTGTGCTGACCCAGACTCCACTCACTTTGTCGGTTACCATTGGACAACCAGCCTCCATCTCTTGCAAGTCAAGTCAGAGCCTCTTAGATAGTGATGGAAAGACATTTTTGAATTGGTTGTTTCAGAGGCCAGGCCAGTCTCCAAAGCGCCTAATCTATCTGGTGTCTAAACTGGACTCTGGAGTCCCTGACAGGTTCACTGGCAGTGGATCAGGGACAGATTTTACACTGAAAATCAGCAGAGTGGAGGCTGACGATTTGGGAGTTTATTATTGCTGGCAAGGTACACATTTTCCTCAGACGTTCGGTGGAGGCACCAAGCTGGAAATCAAA

**Light chain: Amino acid sequence (131aa)**

Signal peptide-FR1-CDR1-FR2-CDR2-FR3-CDR3-FR4

MSPAQFLFLLVLWIRETNGDVVLTQTPLTLSVTIGQPASISCKSSQSLLDSDGKTFLNWLFQRPGQSPKRLIYLVSKLDSGVPDRFTGSGSGTDFTLKISRVEADDLGVYYCWQGTHFPQTFGGGTKLEIK
